# Climate‐Driven Shifts in Wind Energy Potential Across Central Asia Based on a Multi‐Model Ensemble and Tier‐Based Assessment

**DOI:** 10.1002/gch2.202500503

**Published:** 2026-09-12

**Authors:** Parya Broomandi, Mehdi Bagheri, Sonny Irawan, Aram Fathian, Ali Mozhdehi Fard, Dorna Gholamzade Ledari, Michael Leuchner, Jong Ryeol Kim

**Affiliations:** ^1^ Department of Civil and Environmental Engineering School of Engineering and Digital Sciences Nazarbayev University Astana Kazakhstan; ^2^ Department of Electrical and Computer Engineering School of Engineering and Digital Sciences Nazarbayev University Astana Kazakhstan; ^3^ Department of Petroleum Engineering School of Mining and Geoscience Nazarbayev University Astana Kazakhstan; ^4^ Water, Sediment, Hazards, and Earth‐surface Dynamics (waterSHED) Lab Department of Geoscience University of Calgary Calgary Alberta Canada; ^5^ Physical Geography and Climatology Department of Geography RWTH Aachen University Aachen Germany

**Keywords:** central Asian wind energy potential, climate risk for wind energy, CMIP6 climate models, wind energy planning, wind farm siting, wind speed variability

## Abstract

Reliable assessment of future wind energy resources is crucial forsustainable energy planning in Central Asia (CA), where wind patterns arehighly sensitive to climate change. This study assesses historical andprojected 100 m wind speed during 1992–2100 using multi‐ensemble outputs fromthe Coupled Model Intercomparison Project Phase 6 (CMIP6) under SharedSocioeconomic Pathways (SSP2‐4.5 and SSP5‐8.5), bias‐corrected using EuropeanCentre for Medium‐Range Weather Forecasts (ECMWF) ERA5 reanalysis. We developeda novel ranking framework integrating seasonal variability, Weibull‐based windcharacterization, and exceedance probabilities, while accounting for scenariosensitivity. Historically, seasonal wind speeds increased during spring andsummer, particularly in Kyrgyzstan and Kazakhstan; however, SSP5‐8.5projections indicate widespread declines, especially in fall. Seasonal shiftsalso emerge, with peak wind months moving from spring to winter or summer.These changes produce clear differences in site stability: Kazakhstan hosts∼23% of Tier 1 sites, Kyrgyzstan 〈2%, while Turkmenistan and Uzbekistan aredominated by Tier 5 sites requiring case‐specific evaluation. Overall, the results highlight strong spatial contrasts in windenergy potential across CA and provide a robust basis for prioritizinginvestments, reducing climate‐related planning uncertainty, and supportingresilient, climate‐adaptive wind energy strategies.

## Graphical abstract

This study presents a climate‐informed framework for assessing future wind energy potential across Central Asia using bias‐corrected Coupled Model Intercomparison Project Phase 6 (CMIP6) 100 m wind‐speed projections. Integrating Weibull characterization, seasonal variability, exceedance probabilities, and scenario sensitivity, the framework identifies regional disparities, seasonal shifts, and site stability, supporting risk‐informed wind energy investment and resilient climate‐adaptive planning.

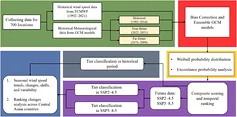

## 1 Introduction

Human activities have caused unprecedented changes in the global climate through greenhouse gases (GHG) emissions [1]. Therefore, switching to low‐carbon renewable energy sources is required to cut carbon emissions and limit this phenomenon. In the energy transition, wind power has a potential important role, but despite its important role in combating and mitigating climate change, it is highly dependent on the climate and its variability. The available wind energy potential depends on different parameters [2, 3], such as wind speeds which are proportional to the wind speed cubed, and small fluctuations lead to more significant changes in wind power. Consequently, it is essential to evaluate the future changes in wind energy potential caused by climate change as a main driver of its fluctuations to manage upcoming investments [3, 4, 5, 6, 7, 8, 9, 10, 11, 12, 13, 14, 15].

For example, a European research study analyzed the impact of climate change on wind power generation using two climate scenarios (RCP 4.5 and RCP 8.5) for 2048–2098, compared to 1970–2020 [7]. The findings showed that most models predicted either no change or an increase in wind drought duration, particularly during spring. Northern regions might experience a slight increase in wind energy droughts, though not all models agreed on these changes. For summer, models consistently showed a decrease in wind droughts over Spain and much of Turkey, especially under the RCP 8.5 scenario, with potential reductions exceeding 100 days [7]. This highlights the seasonal dependencies and the spatial heterogeneity of wind resource changes across Europe. Moreover, onshore wind energy resources in North America were assessed using CMIP6 SSP2–4.5 and SSP5–8.5 climate scenarios for the periods 2051–2060 and 2091–2100, compared to the base period of 2005–2014 [15]. In both scenarios, onshore wind power density in the United States and Canada is predicted to decline. Under SSP2–4.5, a localized decrease of up to 30% is projected for the Eastern United States, particularly in the Midwestern states south of the Great Lakes, including Wisconsin, Illinois, Michigan, Indiana, and Ohio. Under SSP5–8.5, an overall reduction of 15% is expected, with declines reaching as much as 40% in certain northern regions, such as Quebec and Nunavut in Canada, and Alaska in the United States. Conversely, significant increases are projected in Central America and southern Mexico (up to 30%), Hudson Bay (up to 25%), and northern Mexico and Texas (up to 15%) [15].

As climate change emerges as a key driver influencing the variability of wind energy resources, understanding the current and future wind energy potential in CA becomes imperative, given its diverse geographic landscape that significantly influences its wind energy potential and substantial untapped opportunities [16]. Recent regional assessments confirm that CA possesses considerable renewable energy potential, particularly for wind and solar resources, although deployment remains limited due to infrastructure, financial, and policy barriers [16]. Among these nations, Kazakhstan demonstrates the highest potential for wind power, estimated at 354 000 MW [17]. A more conservative assessment identifies a technically feasible capacity of 1000–2000 MW and an economically feasible potential of 250 MW [18]. The country's wind power generation potential has been quantified as 2 billion GWh gross, 1.82 million GWh technically feasible, and 1710 GWh economically feasible [19].

CA possesses significant wind energy potential, yet its development is hindered by several key challenges, such as seasonal wind variability, infrastructure limitations, high costs, and policy gaps that limit the effective utilization of these resources. Seasonal variability in wind availability complicates consistent energy production, with higher wind speeds in winter and lower speeds in summer, particularly in regions like the Dzungarian Gates [16, 20]. Limited transmission infrastructure prevents the integration of remote high‐potential wind sites into the grid, while high initial investment costs, including reliance on imported equipment and lack of local manufacturing, further constrain development [16, 21, 22]. Additionally, inconsistent regulatory frameworks and the absence of clear incentives, such as feed‐in tariffs and tax exemptions, discourage private sector participation [23]. Recent energy system analyses further emphasize that large‐scale renewable deployment in Kazakhstan will require substantial grid modernization, storage integration, and infrastructure upgrades to maintain system stability under higher renewable penetration scenarios [16, 24]. Addressing these barriers through improved infrastructure, supportive policies, and regional cooperation is essential to unlocking the region's vast wind energy potential.

Despite these efforts, existing wind‐resource assessments in CA have several limitations. Many studies rely primarily on mean wind‐speed estimates, focus on historical observations, or evaluate wind potential for a single period without considering long‐term climate‐driven variability. Such approaches may overlook seasonal fluctuations, scenario‐dependent changes, and the evolving spatial stability of wind resources under future climate conditions. These limitations highlight the need for integrated, scenario‐based frameworks that can capture both temporal variability and long‐term resilience of wind resources.

The goal of this research, leveraging multi‐CMIP6 GCM ensemble predictions, is to address four key objectives: (1) Analyzing the impact of climate change on wind energy potential by examining trends in wind speed strength and stability across different seasons and months under SSP2–4.5 (moderate emissions) and SSP5–8.5 (high emissions) scenarios; (2) Ranking and comparing locations based on wind energy potential across different time periods and climate scenarios; (3) Identifying plausible locations vulnerable to the combined threats of declining wind availability and increasing climate variability; and (4) Determining optimal locations for wind energy development by selecting sites that demonstrate long‐term wind stability and resilience across climate projections. Additionally, this research evaluates whether areas previously overlooked may become viable for new wind energy installations or whether regions with an already well‐developed wind energy industry may see their wind energy potential endangered or, conversely, enhanced. In any case, studying the evolution of wind energy potential is fundamental for the planning of future wind energy infrastructure and achieving CA's renewable energy goals [3]. Overcoming these barriers is crucial to leveraging CA's vast wind resources to meet renewable energy goals, enhance energy security, and reduce reliance on fossil fuels.

This study contributes to existing literature in three key ways. First, it integrates bias‐corrected CMIP6 projections with seasonal wind‐speed variability and exceedance probability analysis to provide a more comprehensive assessment of wind energy potential. Second, it introduces a multi‐period and multi‐scenario ranking framework that explicitly accounts for temporal evolution and emissions‐driven uncertainty. Third, it identifies climate‐resilient and vulnerable locations across CA, offering a decision‐support tool for long‐term wind energy planning.

Building on these contributions, the proposed framework advances regional wind‐energy assessment by introducing a multi‐scenario, multi‐period ranking framework that integrates CMIP6‐based future projections with seasonal wind‐speed variability, exceedance probabilities, and a tier‐classification scheme. Unlike previous studies that rely primarily on mean wind speeds or single‐period evaluations, our framework simultaneously evaluates historical (1992–2021), near‐future (2022–2051), and far‐future (2071–2100) conditions under SSP2–4.5 and SSP5–8.5. The approach captures both temporal evolution and scenario sensitivity of wind resources, offering a region‐specific decision‐support tool for sustainable energy planning across CA—a region where comprehensive, scenario‐driven analyses remain scarce.

## 2 Methodology

### 2.1 Study Area

**FIGURE 1:**
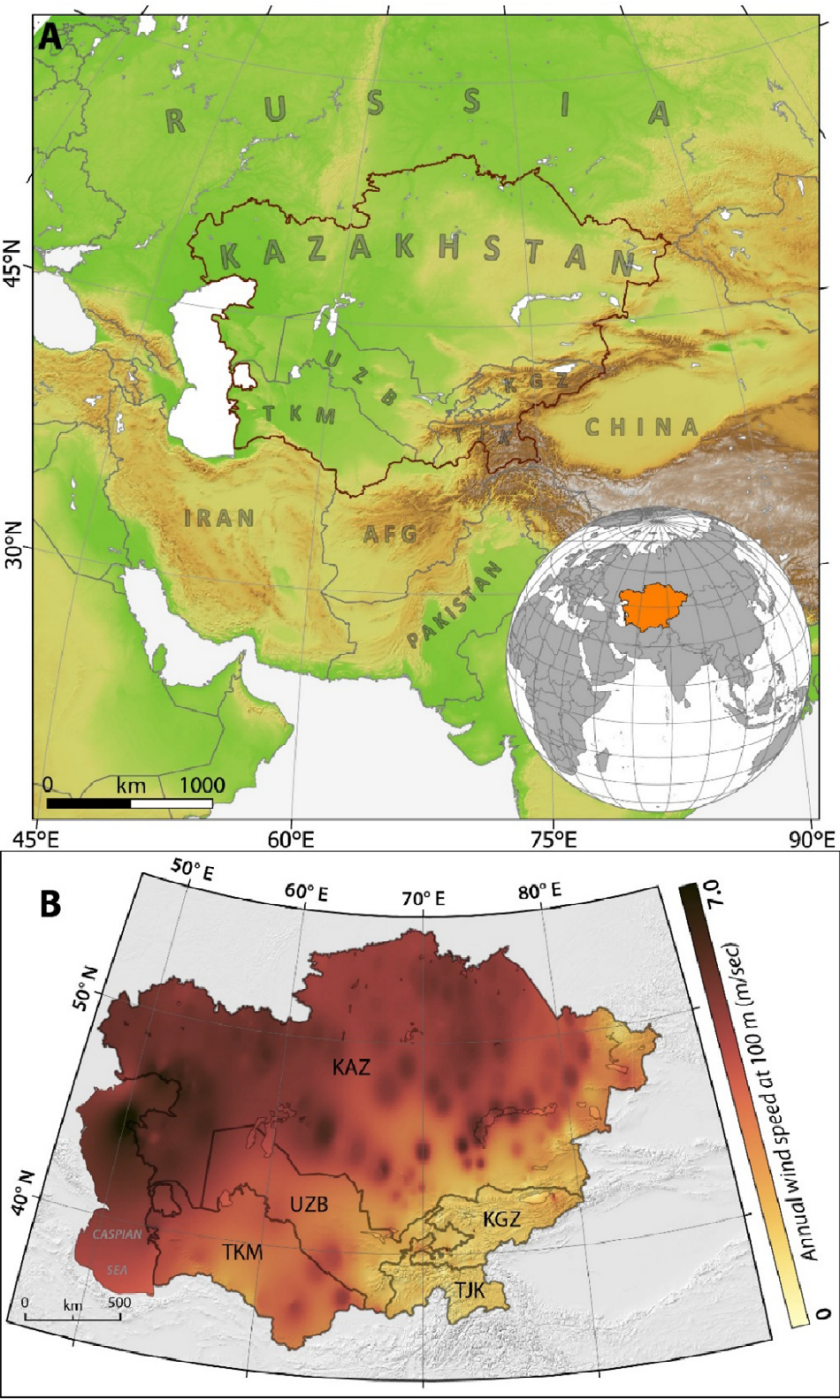
(A) The area of interest, CA consists of Kazakhstan, Kyrgyzstan, Tajikistan, Turkmenistan, and Uzbekistan. (B) Annual mean wind speed at 100 m between 1922 and 2021.

CA spans approximately 4 million km^2^ between 50° and 90° East longitude and 20° to 60° North latitude, comprising five countries: Kazakhstan, Kyrgyzstan, Tajikistan, Turkmenistan, and Uzbekistan [25] (Figure 1). The region is geographically and climatically diverse, broadly divided into northern temperate and southern arid‐to‐semi‐arid zones [26]. These distinctions significantly influence wind regimes and renewable energy potential across the region. As shown in Figure 1, the northern part of CA is primarily represented by Kazakhstan's steppe regions, while the southern part includes the mountainous areas of Kyrgyzstan and Tajikistan and the arid lowlands of Turkmenistan and Uzbekistan. These geographic differences shape regional wind circulation patterns and seasonal wind variability across the study area.

Wind profiles in CA are shaped by topography, synoptic circulation, and terrain‐driven effects such as channeling and turbulence. Among the five countries, Kazakhstan exhibits the most favorable wind speed conditions, especially in the Dzungarian Gates, where wind speeds have been recorded in the range of 7 to 9 m/s, making it one of the region's most promising wind corridors [18]. Other regions, such as the Mangystau Region, Karatau Mountains, and Chu‐Ili Mountains, are also identified as high‐potential sites, although detailed speed measurements for these zones are not consistently reported [27].

In Kyrgyzstan, wind regimes are shaped by mountainous terrain and site‐specific circulation effects. Although most areas experience variable wind conditions, certain exposed alpine sites have recorded wind speeds of up to 8 m/s, which, according to local assessments, can support turbine operation at full capacity for up to 5500 h per year [16]. Estimates of wind potential in Uzbekistan and Turkmenistan vary significantly between sources—ranging from 100 MW to over 520 GW in Uzbekistan alone—reflecting the lack of standardized methodologies and systematic measurement efforts [17, 20].

### 2.2 Data Source

The present research obtained daily meteorological data from the European Centre for Medium‐Range Weather Forecasts (ECMWF) ERA5 reanalysis dataset [28], which is generated by the ECMWF integrated forecasting system (IFS CY41R2) through four‐dimensional data assimilation of global observations. ERA5 is a widely used global reanalysis product that provides spatially and temporally consistent atmospheric variables and has been extensively evaluated for climate and wind resource studies [29]. In this study, wind speed fields at 10 m height (m/s) were extracted at 700 ERA5 locations distributed across CA, covering urban, suburban, and rural regions, for the period 1992–2021. In this study, each “location” corresponds to a predefined geographic coordinate across CA, for which meteorological data were extracted from the nearest ERA5 grid point. A total of 700 locations were selected based on available geographic datasets and city‐level information across the five Central Asian countries (Kazakhstan, Kyrgyzstan, Tajikistan, Turkmenistan, and Uzbekistan), ensuring coverage of urban, suburban, and rural areas. The World Cities Database and World Population Review were used to obtain the spatial distribution and geographic coordinates of populated areas.

GCM outputs from CMIP6 were obtained from the Copernicus Climate Change Service (C3S) to evaluate the effects of climate change on key atmospheric variables across CA. The CMIP6 model outputs used in this study were available at a monthly temporal resolution, which is the standard temporal frequency for multi‐model ensemble climate projections. The analysis included all CMIP6 models available in common across the historical (1992–2014), SSP2–4.5, and SSP5–8.5 experiments, ensuring consistent temporal and scenario coverage. A total of 21 CMIP6 global climate models (GCMs) satisfied these criteria and were included in the analysis, and the complete list of models is provided in Table S1. Two future timeframes were examined—the near‐future (2022–2051) and the far‐future (2071–2100)—to capture both mid‐century and end‐century changes (Table S1). Seasonal averages were calculated using the standard climatological definition: winter (December–February), spring (March–May), summer (June–August), and fall (September–November). The SSP2–4.5 and SSP5–8.5 pathways were selected because they represent the most policy‐relevant and contrasting futures: SSP2–4.5 reflects a stabilization scenario consistent with intermediate mitigation efforts, while SSP5–8.5 corresponds to a high‐emission, fossil‐fuel‐intensive trajectory that defines the upper bound of potential climate impacts [30]. This dual‐scenario approach enables evaluation of both moderate and extreme warming responses within the same modeling framework.

In this study, interpolation to a spatial resolution of 0.1° × 0.1° was performed using the inverse distance weighting (IDW) algorithm solely for visualization purposes, generating smoother spatial representations. All analytical procedures were conducted using the datasets at their original spatial resolutions—ERA5 (0.25°) and CMIP6 at their respective native resolutions, which vary by model.

### 2.3 Climate Change: Statistical Analysis of GCMs and Different Scenarios

Future climate projections for CA were derived from multiple GCMs using two climate scenarios from the CMIP6 archive: SSP2–4.5 and SSP5–8.5 [30].

By utilizing observed data, bias correction was employed to correct modeling discrepancies in the outputs of GCMs. In this study, bias correction was applied to the 10 m wind speed data, which represents the standard reference height consistently available in both CMIP6 model outputs and the ERA5 reanalysis dataset. Applying bias correction at the native model level avoids introducing additional uncertainties that may arise from correcting wind speeds after vertical extrapolation to higher altitudes. A hybrid semi‐parametric method was deployed to analyze the CMIP6 GCMs data from 1992 to 2100. The bias‐correction transfer functions were derived from the historical overlap period (1992–2014) between CMIP6 simulations and ERA5 reanalysis data and were subsequently applied to the future projections (2022–2100). This method adjusts the statistical distribution of modeled wind speeds to match ERA5 data while preserving long‐term climate change signals [31]. In practice, the procedure aligns the statistical distribution of modeled wind speeds with the ERA5 data while preserving the long‐term climate change signals simulated by the GCMs. This approach assumes that differences between observed and modeled climate variables remain constant throughout time, enabling future projections to be made directly from historical data using a transfer function [31]. The discrepancies in results generated by different GCMs were addressed using an unweighted approach (one model, one vote) [31]. Each GCM was bias‐corrected individually before computing the ensemble mean, and ensemble averages were then generated by assigning equal weighting to each model. After bias correction, wind speeds were subsequently extrapolated to 100 m height to represent typical wind turbine hub heights used in modern wind energy systems. Additionally, the method ensures consistent adjustment of distribution functions across the entire time series, which is essential for reducing systematic biases while retaining physically meaningful variability in the GCM outputs [31].

This study employed all CMIP6 global climate models (GCMs) available in common across these experiments to ensure consistent temporal and scenario coverage. Using the full overlapping ensemble avoids structural bias introduced by selective model sampling and provides a comprehensive representation of plausible future wind‐climate conditions. All models included have been evaluated within the CMIP6 framework for large‐scale circulation and surface‐wind performance [32]. By applying the ensemble mean of these models, this study reduces individual‐model noise and internal variability, yielding a robust central estimate of projected changes while acknowledging that some uncertainty remains due to inter‐model spread and regional downscaling limitations. Moreover, to statistically assess the performance of the model in simulating the interest variables, we computed the bias and root mean square error (RMSE) [33].

Trend significance was evaluated using the nonparametric Mann–Kendall test, a widely applied method for detecting monotonic trends in climate time series. A significance threshold of 5% (*p*‐value < 0.05) was adopted to identify statistically significant trends [34, 35, 36, 37]. All slope values reported in this study represent changes in wind speed and are expressed in units of meters per second (m/s).

### 2.4 Wind Energy Ranking and Tier Classification Framework

To assess the long‐term wind energy suitability of sites across CA, we developed a multi‐criteria, climate‐informed ranking system incorporating seasonal wind variability, exceedance probabilities, and scenario‐based projections.

**FIGURE 2:**
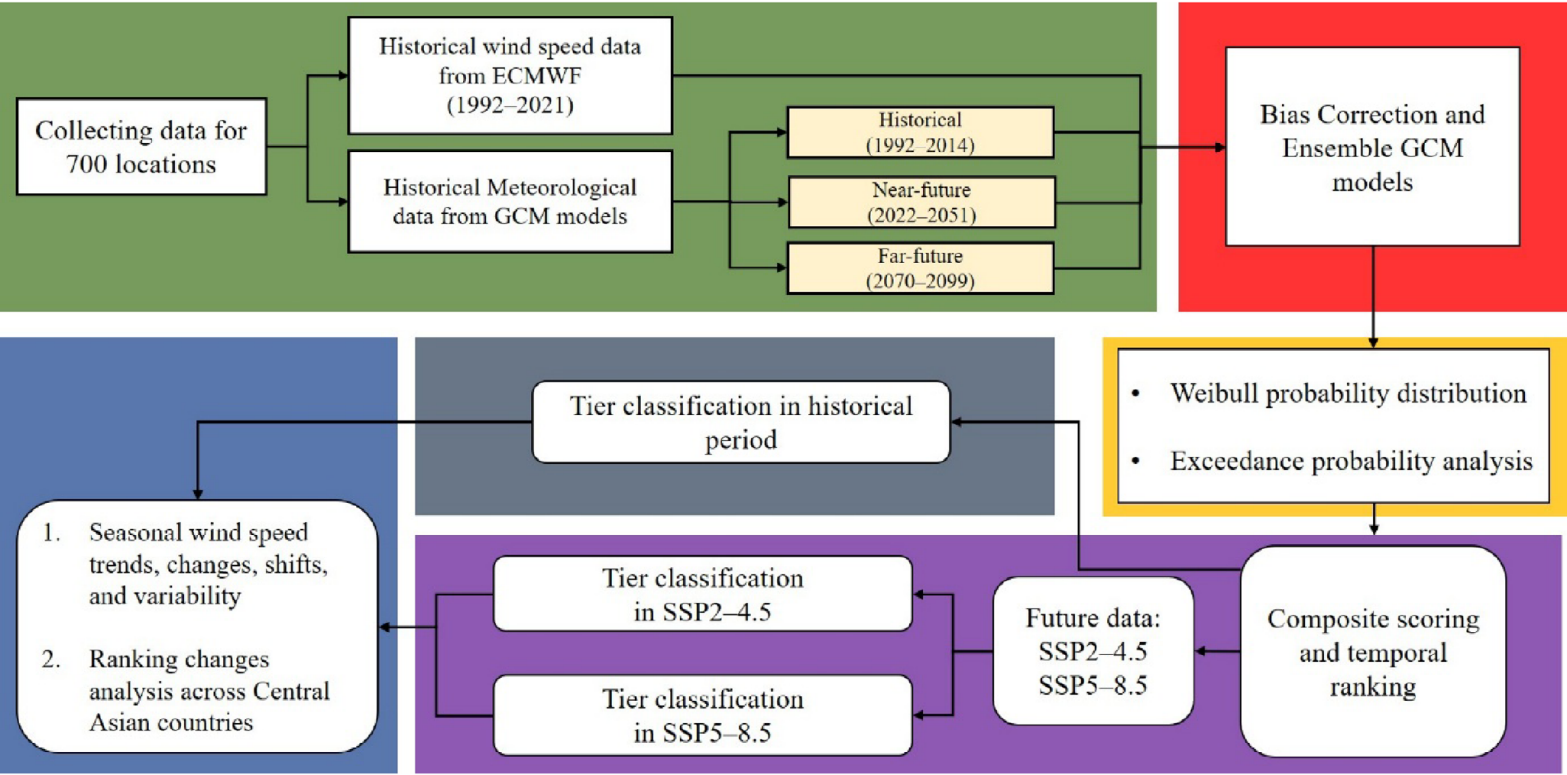
Overview of the methodological framework used in this study. The workflow illustrates the sequence from data collection at 700 locations using ERA5 and CMIP6 datasets, through bias correction and ensemble processing, to wind speed characterization using Weibull probability distribution and exceedance probability analysis. The resulting metrics are integrated into a composite scoring system to generate temporal rankings and tier classifications under SSP2–4.5 and SSP5–8.5 scenarios for historical, near‐future, and far‐future periods.

Rankings were generated independently for each site and time period under historical, 1992–2021, near‐future, 2022–2051, and far‐future, 2071–2100, intervals using data from SSP2–4.5 and SSP5–8.5 scenarios. The overall methodological framework adopted in this work is summarized in Figure 2, illustrating the workflow from data collection and bias correction to wind speed distribution analysis, ranking, and tier classification under both historical and future climate scenarios (SSP2–4.5 and SSP5–8.5).

#### 2.4.1 Wind Speed Standardization and Data Structuring

To estimate wind behavior at standard turbine hub height (assumed here as 100 m), all wind speeds were extrapolated using the power law (Hahmann et al., 2022):

(1) $$V_{h}=V_{r}\;{\left(\frac{h}{r}\right)}^{\alpha }$$

where *V_h_* is the wind speed at hub height *h*, *V_r_* is the observed speed at reference height *r* (10 m), and *α* is the wind shear exponent A constant exponent value of 0.143 was applied, representing a neutrally stable atmosphere, following common practice in regional‐scale wind‐resource assessments [38]. Although the wind‐shear exponent varies with atmospheric stability and surface roughness—typically higher over rough terrain (e.g., forests) and lower over smooth surfaces (e.g., deserts)—spatially and temporally resolved stability data were unavailable for this study. Hence, adopting a neutral value ensures consistency across all grid cells and facilitates comparison with previous large‐scale assessments. Future work will incorporate dynamically varying shear exponents derived from planetary boundary‐layer stability diagnostics to improve site‐level precision [38].

### 2.5 Weibull‐Based Wind Speed Fitting and Scale Parameter Estimation

To characterize the wind speed frequency distribution at each site, we applied the two‐parameter Weibull probability distribution [39, 40]. In this study, the analysis focused on the Weibull scale parameter (*c*), which reflects the characteristic wind speed and is directly related to the mean wind energy potential. Although the mean wind speed of a Weibull distribution depends on both the scale (*c*) and shape (*k*) parameters, the scale parameter is widely used in wind‐resource assessments as a robust indicator of wind magnitude and wind energy potential Seguro and Lambert, 2000 [41, 42, 43].

The focus on the scale parameter allows consistent comparison of wind resource changes across different time periods and climate scenarios while avoiding additional uncertainty associated with estimating multiple distribution parameters from projected datasets. The scale parameter was estimated for each point and period (historical, near‐future, and far‐future). The resulting parameter values were used to quantify changes in the characteristic wind regime and to support the ranking and tier classification described in the following sections.

The Weibull shape parameter (*k*), which describes the variability of the wind speed distribution, was not directly used in the ranking framework because wind variability is already captured in the composite scoring system through the explicit wind variability metric and exceedance probability analysis. This approach prevents overlapping representations of wind variability while maintaining a physically meaningful characterization of wind resource quality.

#### 2.5.1 Exceedance Probability Analysis

To capture turbine‐operational reliability, we calculated seasonal exceedance probabilities for each site and time period, reflecting how often wind speeds fall within or beyond key performance thresholds. These thresholds were based on international standards and manufacturer guidelines and are summarized in Table 1 below.

**TABLE 1:** Wind speed operational thresholds and corresponding wind energy potential categories used in ranking analysis [44].

Wind speed range (m/s)	Category	Wind energy potential
<3	Below cut‐in	No power generation
3–7.9	Near cut‐in to low efficiency range	Minimal to limited power generation
8–11.9	Optimal operational range	High power generation (approaching rated capacity)
12–24.9	Strong winds (risk of cut‐out)	High power generation (risky)
≥25	Extreme (cut‐out zone)	No power generation (turbine shut down)

#### 2.5.2 Composite Scoring and Temporal Ranking

A composite performance score was generated for each site in each time period using the following formula:

(2) $$\begin{array}{c} Score=\left(1\times Weibull\;Scale\right)+\left(-1\times Wind\;variability\right)\\ +\left(1\times Prob_{8-11}\right)+\left(-1\times Prob_{>25}\right)+\left(-1\times Prob_{<3}\right) \end{array}$$

The +1 and −1 weights were assigned to reflect the direction of impact:

- +1 weights for beneficial characteristics (e.g., higher scale, optimal exceedance).
–1 weights for undesirable or risky attributes (e.g., extreme wind, high variability).

The scores were then ranked using a dense descending rank function, where a lower rank indicates a higher wind energy suitability. Although this weighting scheme may appear subjective, it follows widely used multi‐criteria decision‐making approaches in wind‐resource assessments [45, 46]. The equal‐magnitude weights (+1/−1) were chosen to maintain neutrality among parameters and to ensure that the scoring reflects the physical influence of each variable rather than arbitrary preference. The relative simplicity of the weighting scheme minimizes potential bias, and moderate adjustments to the assigned weights would not be expected to significantly alter the ranking outcomes, indicating conceptual robustness and transparency of the framework.

#### 2.5.3 Tier Classification for Investment Strategy

To translate site‐level rankings into investment priorities, each location was classified into one of five tiers based on its temporal performance trends and sensitivity to emissions scenarios. This system is grounded in the assumption that both historical performance and future stability are critical to long‐term infrastructure success. Sites were categorized not only based on their baseline suitability, but also on their degree of improvement, decline, or sensitivity to emissions scenarios. The approach assumes that locations with high wind potential and low variability across time and emissions pathways represent the lowest investment risk, while those with volatile or scenario‐dependent behavior may require more adaptive or flexible deployment strategies.

The five‐tier structure was derived using a quantile‐based segmentation of composite scores to ensure objectivity and reproducibility, rather than relying on arbitrary thresholds. Tiered ranking systems have been widely applied in renewable‐energy suitability assessments to facilitate transparent decision‐making and policy interpretation [41, 46]. This approach allows regional and temporal comparisons of site stability while maintaining clarity for planners and investors in identifying resilient, high‐priority wind‐energy zones.

The classification thresholds—summarized in Table 2—were empirically derived from the observed distribution of rank changes across historical, near‐future, and far‐future periods. For instance, a rank variation of ±10 was chosen to identify stable high‐performing sites within approximately the top fifth percentile of temporal consistency, while improvements of ≥+40 and deteriorations of ≥+25 correspond to the upper and lower 10th percentiles, respectively. A scenario sensitivity threshold of ≥±5 ranks between SSP2–4.5 and SSP5–8.5 captures emissions‐driven divergence. These thresholds reflect real behavior within the dataset rather than arbitrary cutoffs. Conceptually, the tiering framework aligns with prior methodologies in wind energy site assessment, including exceedance‐ and variability‐based stability metrics [41, 44]. Each tier corresponds to a distinct investment strategy—from immediate low‐risk deployment to case‐specific evaluation—designed to support adaptive, data‐informed decision‐making under evolving climate conditions.

**TABLE 2:** Tier‐based classification system for wind energy investment planning. The table defines five tiers based on ranking behavior, climate scenario sensitivity, and long‐term performance trends, with tailored strategies for infrastructure deployment and risk management in CA.

Tier	Definition	Classification criteria	Recommended strategy
Tier 1: stable high performers	Consistent top‐ranking sites with minimal variability	Historical rank ≤100 and ≤±10 change in both SSP2–4.5 and SSP5–8.5	Priority large‐scale and low‐risk investment
Tier 2: significant improvers	Rapidly improving sites over time	≥+40 rank improvement in either SSP scenario	Staged investment with monitoring
Tier 3: scenario‐sensitive	Performance varies by emission scenario	≥±5 rank difference between both SSP2–4.5 and SSP5–8.5 (far‐future)	Hybrid/flexible infrastructure needed
Tier 4: consistent decliners	Declining performance under all scenarios	≥+25 rank deterioration in both SSP scenarios	Avoid a wind‐only investment
Tier 5: mixed/unclassified	Unstable or inconsistent behavior	Does not meet criteria for other Tiers	Case‐by‐case evaluation required

## 3 Results and Discussion

### 3.1 Statistical Assessment of Bias Correction Performance

An evaluation of 10 m wind speed over the period 1992–2021 in CA reveals that the raw multi‐ensemble GCM outputs possess a mean positive bias of 1.20 m/s and an associated RMSE of 1.35 m/s. These departures from observations are largely attributable to the coarse spatial resolution of global models, which tend to misrepresent local orographic effects and diurnal wind fluctuations. Upon application of the bias‐correction procedure, the systematic bias is entirely removed (0 m/s), and the RMSE is reduced to 0.42 m/s, with a corresponding reduction in variability (RMSE std decreasing from 0.42 to 0.19 m/s). Table 3 summarizes country‐level bias and RMSE statistics before and after bias correction, including associated standard deviations to reflect spatial variability across the study region. Country‐level statistics further reveal substantial spatial variability prior to correction, with bias means reaching 1.64 m/s in Tajikistan and 1.56 m/s in Kyrgyzstan, and RMSE values up to 1.69 m/s, accompanied by relatively high standard deviations (e.g., RMSE std = 0.45 m/s in Turkmenistan), indicating pronounced regional uncertainty. After bias correction, both bias and its variability are eliminated across all countries, while RMSE values decrease markedly (e.g., 0.29–0.56 m/s), with reduced standard deviations (≈0.10–0.16 m/s), demonstrating improved consistency across the region.

**TABLE 3:** Country‐level bias and RMSE statistics for 10 m wind speed before and after bias correction across CA (1992–2021). Mean and standard deviation (std) values are reported to quantify spatial variability and uncertainty across the study region.

Country	Raw multi‐ensembled GCM models				Bias‐corrected multi‐ensembled GCM models			
	*Bias mean*	*Bias std*	*RMSE mean*	*RMSE std*	*Bias mean*	*Bias std*	*RMSE mean*	*RMSE std*
KG	1.5573	0.2776	1.6062	0.2298	0	0	0.2919	0.1233
KZ	0.7708	0.466	1.0411	0.3114	0	0	0.5627	0.1589
TJ	1.6434	0.4447	1.691	0.4313	0	0	0.3218	0.1179
TM	0.9271	0.6629	1.1273	0.4518	0	0	0.3914	0.1361
UZ	1.4236	0.3205	1.4791	0.2692	0	0	0.2943	0.0969

This reduction in error demonstrates that the correction method not only aligns the ensemble mean more closely with observed interannual variability but also preserves the temporal structure of extremes [47]. Such improvements are critical for downstream impact assessments, ranging from resource estimation and turbine siting to climate‐resilient energy planning, because they enhance confidence in projected wind speed distributions and mitigate the risk of over‐ or underestimating future wind energy potential [47].

### 3.2 Seasonal Wind Speed Trends in CA

**FIGURE 3:**
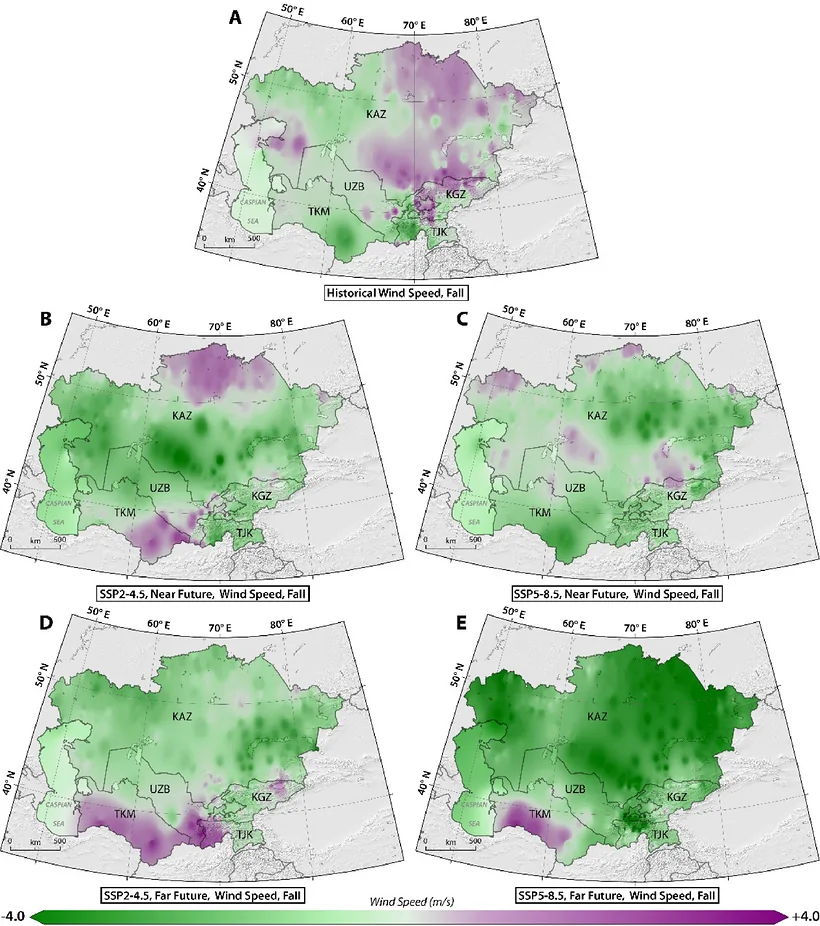
Analysis of spatiotemporal wind speed at 100 m (m/s) in CA during fall from 1992 to 2100 under SSP2–4.5 and SSP5–8.5 climate change scenarios.

**FIGURE 4:**
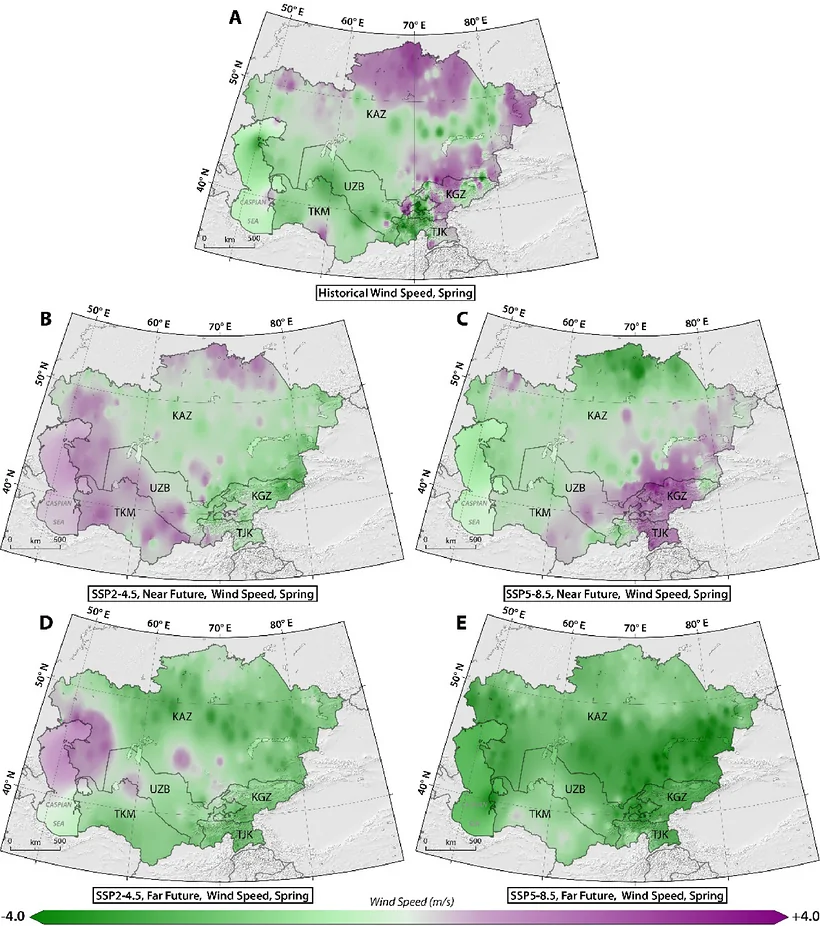
Analysis of spatiotemporal wind speed at 100 m (m/s) in CA during spring from 1992 to 2100 under SSP2–4.5 and SSP5–8.5 climate change scenarios.

**FIGURE 5:**
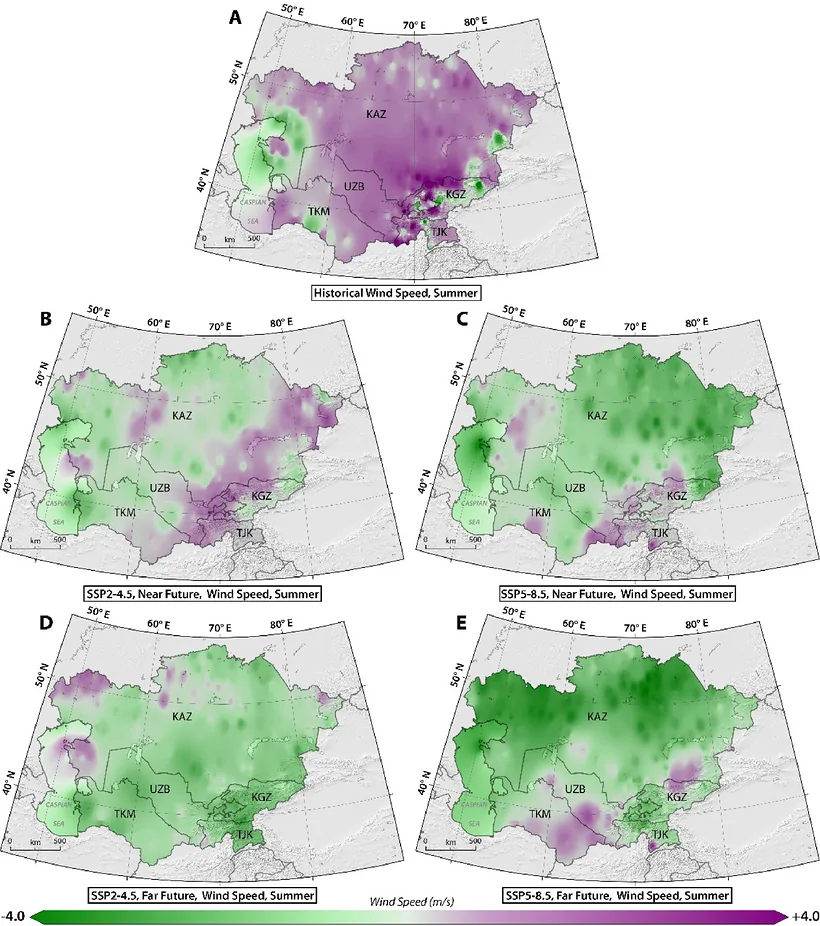
Analysis of spatiotemporal wind speed at 100 m (m/s) in CA during summer from 1992 to 2100 under SSP2–4.5 and SSP5–8.5 climate change scenarios.

**FIGURE 6:**
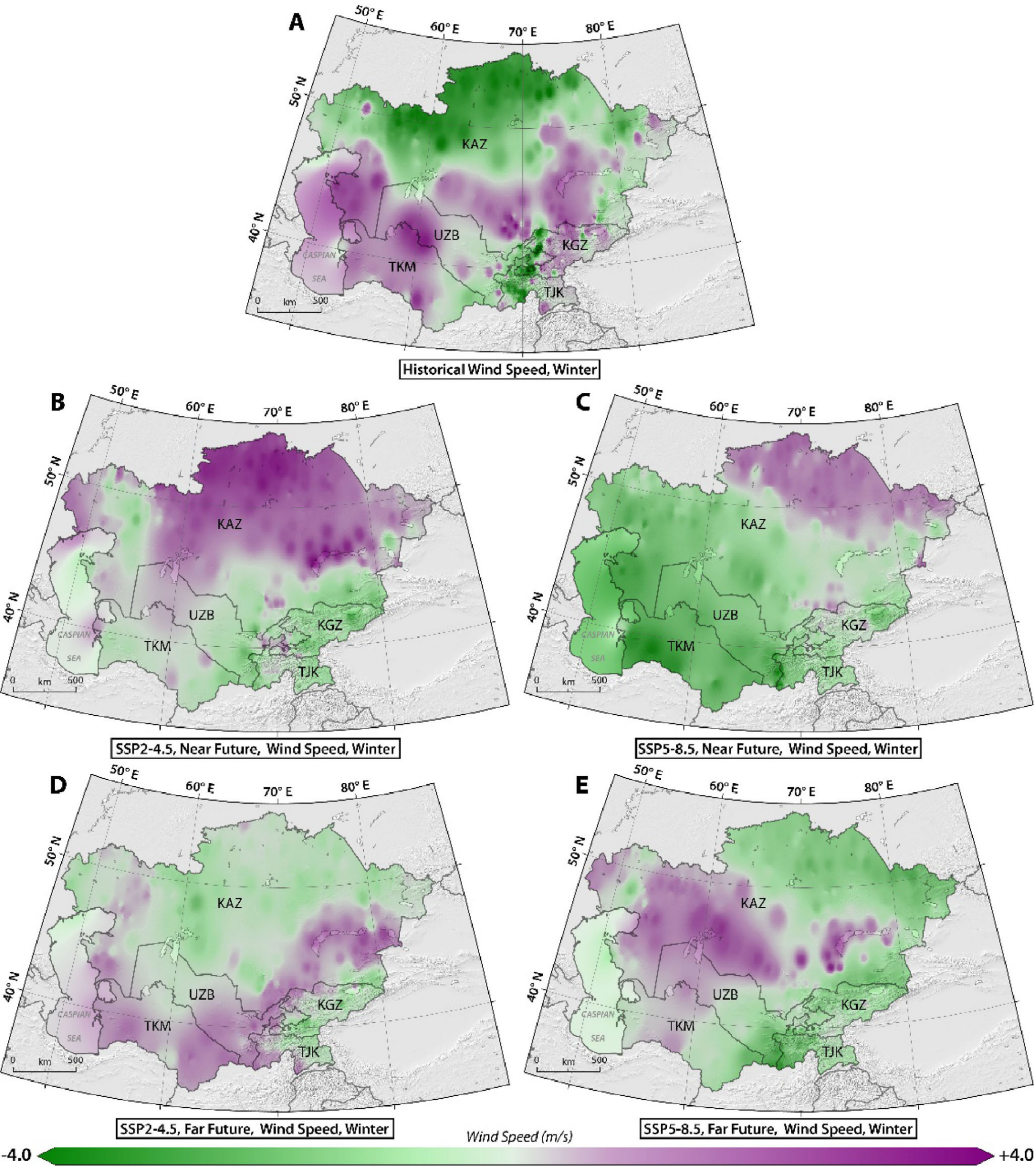
Analysis of spatiotemporal wind speed at 100 m (m/s) in CA during winter from 1992 to 2100 under SSP2–4.5 and SSP5–8.5 climate change scenarios.

Figures 3, 4, 5, 6 show trends in seasonal wind speed changes at 100 m across Central Asian countries during the historical period and under future climate scenarios (SSP2–4.5 and SSP58.5) for both near‐term, 2022–2051, and far‐term, 2071–2100, projections, providing a detailed overview of regional and seasonal variability in projected wind speed changes. All values discussed in this section refer to trend slopes (i.e., rates of change in wind speed), rather than absolute wind speed values. The slope represents the rate of change in seasonal wind speeds, where positive values indicate strengthening winds and negative values indicate weakening trends.

During the historical period, wind speed trends indicate seasonal variability across CA. Spring and summer generally exhibited positive slopes in countries such as Kyrgyzstan (+0.042 and +0.126, respectively) and Kazakhstan (+0.034 and +0.094), suggesting a historical strengthening of seasonal winds. Meanwhile, fall tended toward negative slopes, particularly in Tajikistan (−0.127) and Turkmenistan (−0.095), suggesting a seasonal weakening. This seasonal sensitivity is consistent with findings by Barkanov et al. [48], who identified spring and fall as climate‐sensitive periods for wind energy resources across Europe [48].

In the near‐future (SSP2–4.5), seasonal patterns are more mixed. Summer winds in Kyrgyzstan (+0.046), Tajikistan (+0.096), and Uzbekistan (+0.043) remain slightly positive, but overall, most seasons trend toward neutral or mildly negative slopes, particularly in winter and fall. Under SSP2–4.5, the far‐future shows predominantly negative slope values, indicating a general decline in seasonal wind speeds. Spring and summer slopes turn negative across almost all countries, with values such as −0.136 and −0.170 in Kyrgyzstan and −0.075 and −0.056 in Kazakhstan. However, in the far‐future SSP2–4.5 projections, Tajikistan and Turkmenistan still show minor positive slopes in winter (+0.056 and +0.082, respectively).

In the near future (SSP5–8.5), however, there is still evidence of short‐term strengthening during spring in countries like Kyrgyzstan (+0.125), Uzbekistan (+0.094), and Tajikistan (+0.027). Nevertheless, summer and winter already begin to show weakening trends, particularly in southern CA, with countries like Turkmenistan (−0.014 in summer and −0.235 in winter) and Uzbekistan (−0.121 in summer and −0.102 in winter) experiencing early declines. The far‐future (SSP5–8.5) is characterized by strongly negative slope values, indicating a substantial decline in seasonal wind speeds. Spring and fall suffer the most, with strongly negative slopes across the region: −0.247 and −0.131 in Kyrgyzstan, −0.202 and −0.292 in Kazakhstan, and similar declines elsewhere. Summer also weakens notably in Kazakhstan (−0.186) and Uzbekistan (−0.070). These long‐term declines are consistent with broader CMIP6‐based assessments by Moradian et al. [49], who reported strong wind speed reductions under SSP5–8.5 across Northern Hemisphere mid‐latitudes, driven by altered atmospheric circulation patterns [49].

Seasonally, historical strengthening during spring, summer, and winter is projected to reverse in future periods, with summer and winter emerging as the most vulnerable seasons to future wind speed reductions. In the near future, projections under both SSP2–4.5 and SSP5–8.5 scenarios suggest a more mixed pattern, with some short‐term strengthening observed during spring and fall in northern countries, especially under SSP5–8.5. However, by the far‐future, a substantial weakening becomes dominant, particularly under the high‐emission SSP5–8.5 scenario, where negative slopes prevail across nearly all countries and seasons. This contrasts with findings by Miao et al. [50], who observed the strongest seasonal wind reductions during summer in China, highlighting regional differences in seasonal vulnerability [50]. These results highlight significant spatial and temporal disparities in wind speed trends, emphasizing the need for careful consideration of regional variability when planning future wind energy development strategies in CA.

The projected spatial and temporal variations in wind speeds across CA are plausibly linked to large‐scale circulation responses to warming. Regionally, shifts in the subtropical jet and changes in mid‐latitude westerlies can modify synoptic pressure gradients and thermal contrasts, which in turn can modulate near‐surface winds across CA, including Kazakhstan and Uzbekistan [51]. Additionally, enhanced land warming and land–atmosphere coupling may strengthen seasonal thermal lows in southern areas, altering seasonal wind regimes and contributing to greater interannual variability [52]. These dynamic and thermodynamic factors provide a physical context for the tendency toward reduced wind potential and increased variability under SSP5–8.5, while local influences (topography, land‐use/roughness) likely explain the pronounced sub‐regional heterogeneity, underscoring the value of higher‐resolution downscaling in future work.

It is also worth mentioning that the robustness of the projected wind‐speed changes arises from the use of a multi‐model ensemble mean, which minimizes biases and random fluctuations associated with individual GCMs. Ensemble averaging is a widely adopted practice for climate projections, as it enhances signal‐to‐noise ratios and captures the central tendency of model responses to external forcing [32, 53]. Although this study did not perform model‐by‐model significance testing, the ensemble‐based approach provides a physically consistent representation of regional climate signals, reducing dependence on any single model and offering greater confidence in the projected spatial and seasonal trends across CA.

### 3.3 Seasonal Changes in Wind Speed Categories in CA

**FIGURE 7:**
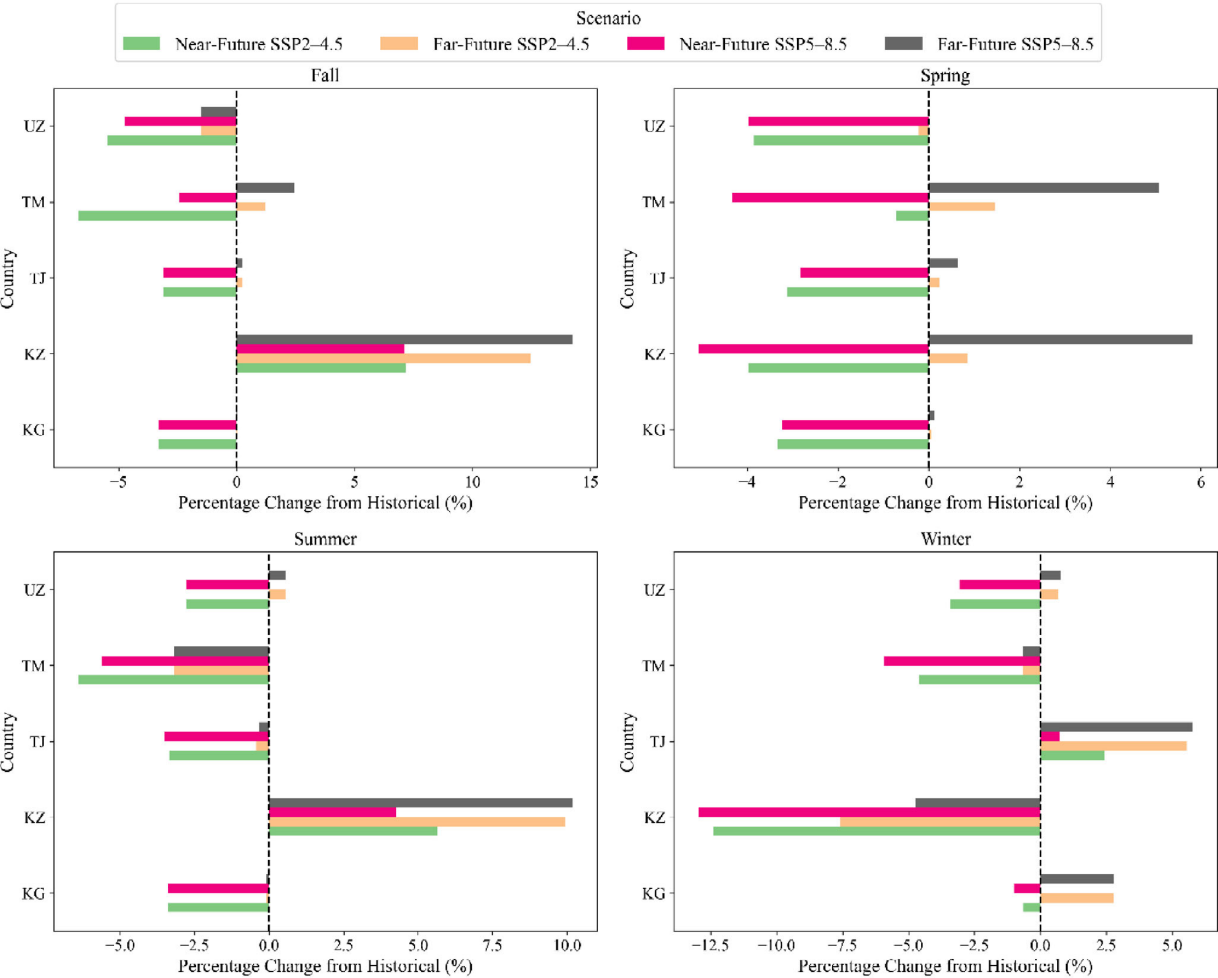
Projected changes in the frequency of calm wind conditions across Central Asian countries under SSP2–4.5 and SSP5–8.5 scenarios for near‐ and far‐future periods.

The analysis of seasonal wind speed category shifts under SSP2–4.5 and SSP5–8.5 reveals significant changes in wind energy potential across Central Asian countries, enabling a nuanced assessment of how climate change scenarios may reshape wind energy productivity. Figure 7 and Figures S1–S3 illustrate seasonal changes in the percentage of locations experiencing different wind speeds (i.e., below turbine cut‐in thresholds) across Kazakhstan (KZ), Kyrgyzstan (KG), Uzbekistan (UZ), Turkmenistan (TM), and Tajikistan (TJ). Bars represent the percentage‐point changes relative to historical baseline conditions (i.e., changes, not absolute values). Projections are shown for each season—fall, spring, summer, and winter—across two future time frames: near‐future, 2022–2051, and far‐future, 2071–2100, under both SSP2–4.5 and SSP5–8.5 scenarios (see Figure 7; Figures S1–S3). In spring, the direction of change appears to be influenced more strongly by the projection horizon than by the emissions scenario, with near‐future projections generally showing negative changes while far‐future projections tend to shift toward neutral or slightly positive values across several countries. This pattern suggests that spring wind resources may experience short‐term weakening followed by partial recovery later in the century, highlighting the importance of considering multiple planning horizons when evaluating long‐term wind energy investments in CA.

Across most regions, the frequency of Calm (Below Cut‐in) wind shows positive changes (i.e., increases relative to historical conditions) in future projections, especially under SSP5–8.5. In Kyrgyzstan, for example, calm winds in fall increase by +0.03% in the far‐future. Tajikistan shows a more noticeable change, with calm winds rising by +0.64% in spring under SSP5–8.5. In contrast, Uzbekistan shows no increase in calm conditions, suggesting better preservation of wind activity near or above cut‐in speeds. The observed increase in calmer conditions aligns with projections by Obahoundje et al. (2024) for West and Central Africa, where climate change similarly leads to increased frequencies of low‐wind‐speed events (Obahoundje et al., 2024). Shifts toward Marginal (near cut‐in) wind conditions are more dramatic. In Kyrgyzstan's fall season, marginal wind frequencies increase by +94.52% under SSP5–8.5, while in Tajikistan, spring shows a +89.47% increase. This shift suggests that more hours fall just around the turbine operational threshold, potentially resulting in lower and more variable energy output (see Figure S1). These findings are consistent with the work of Zuo et al. [54], who reported an increased risk of renewable energy droughts in China under SSP5–8.5, linked to higher variability and reduced operational wind speed conditions [54].

Simultaneously, the proportion of moderate (low efficiency) wind speeds declines significantly. For example, Kyrgyzstan in fall sees a −65.14% drop, and Tajikistan in spring declines by −63.64%. Uzbekistan in winter experiences a −45.95% reduction in moderate wind conditions. These changes reflect a contraction of moderately productive wind ranges in several countries and seasons (see Figure S2). Most critically, the dataset includes optimal (high efficiency) wind conditions, representing ideal wind speeds for turbine output. These show sharp declines under SSP5–8.5. In Kyrgyzstan's winter season, optimal wind speeds are projected to drop by −100%, effectively eliminating ideal wind conditions during that period. The same −100% loss is seen in Kazakhstan (fall), suggesting these regions may experience full seasonal loss of peak wind productivity under high‐emission futures. In contrast, Meza‐Carreto et al. [55] reported slight increases in favorable offshore wind conditions for Mexico under SSP5–8.5, indicating that inland regions may be more vulnerable to climate‐induced losses of optimal wind speeds [55]. Even under the moderate SSP2–4.5 scenario, some locations show increases in optimal winds—like Kazakhstan in spring (+400%)—but this reverses under SSP5–8.5, emphasizing the sensitivity of wind resources to emission trajectories (see Figure S3).

While Tajikistan, Uzbekistan, and Turkmenistan generally exhibit low baseline frequencies of optimal wind conditions, the declines in other categories suggest these countries are also vulnerable to deteriorating wind quality. Turkmenistan, however, remains relatively stable, with changes in optimal wind speeds being minor compared to Kyrgyzstan or Kazakhstan. Overall, SSP5–8.5 accelerates adverse shifts across all categories, pushing regions toward calmer, lower‐efficiency wind conditions. Kyrgyzstan and Tajikistan face rising calm and marginal wind events, while Uzbekistan and Turkmenistan appear more resilient, showing minimal changes in calm wind frequencies and smaller losses in optimal conditions. These findings underscore the importance of strategic adaptation in wind infrastructure across CA. Planners may need to invest in turbine technologies optimized for low‐speed operation, enhance seasonal energy storage, and prioritize high‐resolution wind mapping to identify remaining optimal sites. Expanding the spatial and temporal resolution of future analyses will also be essential to detect localized high‐efficiency wind zones that may not be fully captured in this dataset.

### 3.4 Seasonal Shifts in Wind Speed in CA

**FIGURE 8:**
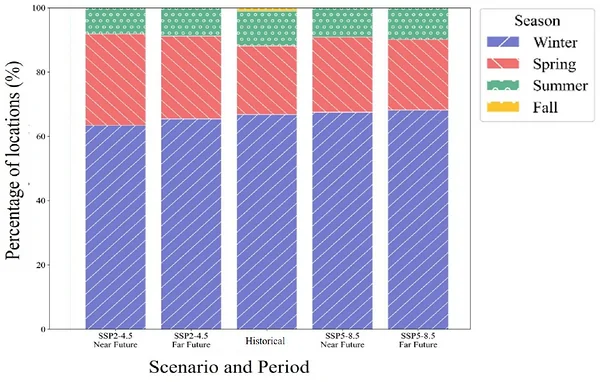
Seasonal wind dominance in Kazakhstan across historical, near‐future, 2022–2051, and far‐future, 2071–2100, periods under SSP2–4.5 and SSP5–8.5 scenarios.

To evaluate how the dominant wind season shifts under different climate scenarios, the percentage of locations dominated by each season (winter, spring, summer, fall) was quantified for each country, scenario, and time frame in terms of absolute percentage distribution, while reported differences represent changes relative to historical conditions (see Figure 8; Figures S4–S7). These plots provide a clear comparison of seasonal dominance distributions across historical, near‐future, and far‐future periods, enabling a clear interpretation of net seasonal changes.

Moreover, to assess the direction and structure of these shifts, season‐to‐season transition matrices were constructed. These country‐specific matrices display the proportion of locations transitioning from one dominant season to another between historical and future periods. This approach captures not only the magnitude but also the underlying directionality of seasonal change (e.g., spring‐dominant areas shifting to winter dominance), offering valuable insight into the dynamics of seasonal rebalancing under climate change (see Figure 8; Figures S4–S7).

**FIGURE 9:**
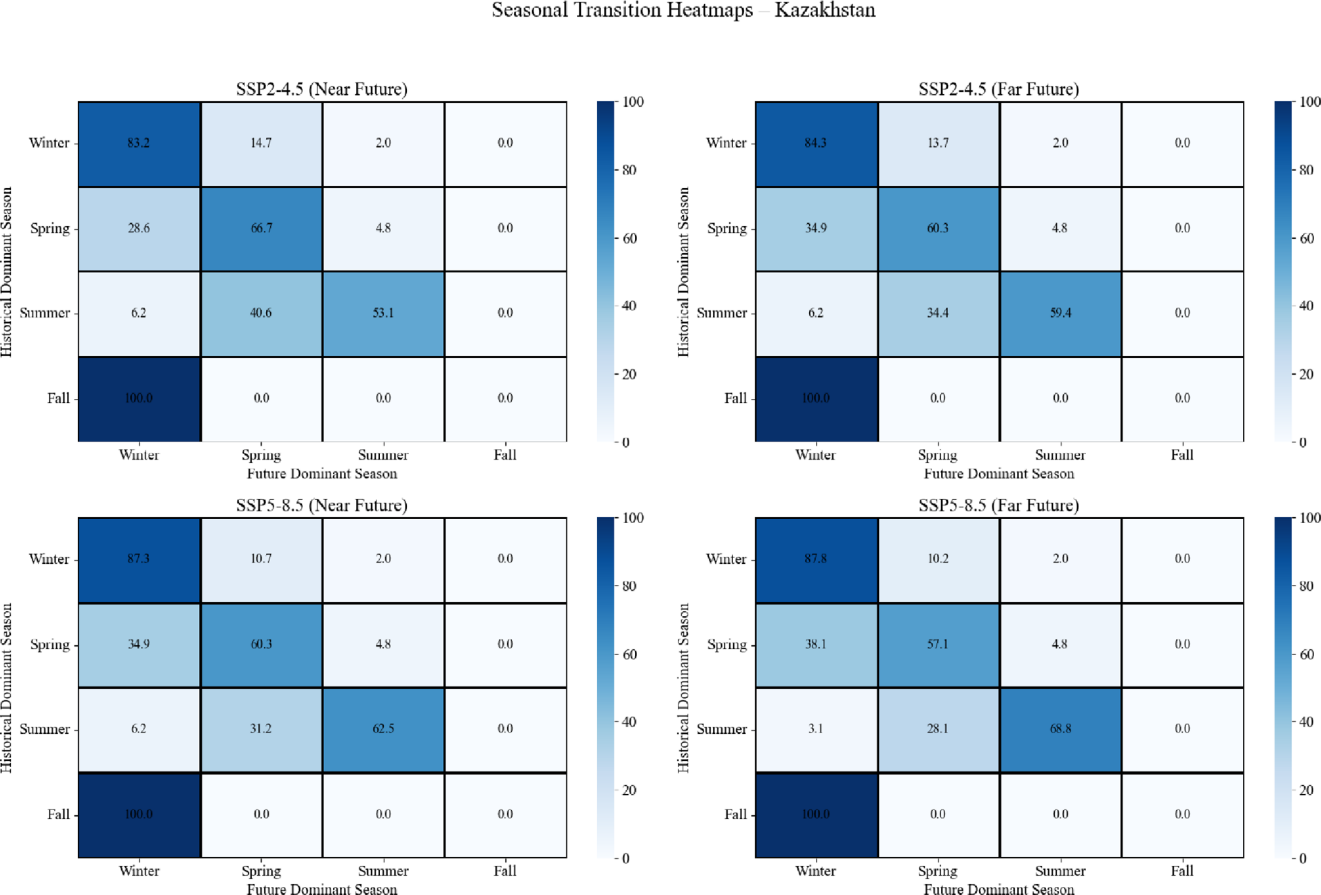
Dominant season transition matrices for Kazakhstan under SSP2–4.5 and SSP5–8.5 scenarios for near‐future, 2022–2051, and far‐future, 2071–2100, periods.

The analysis of seasonal wind dominance across Kazakhstan, Turkmenistan, Kyrgyzstan, Uzbekistan, and Tajikistan under the SSP2–4.5 and SSP5–8.5 scenarios reveals substantial shifts in the intra‐annual distribution of wind resources, with a marked transition toward winter dominance, especially under SSP5–8.5 in the far‐future period, 2071–2100. Among all countries, Tajikistan shows the most significant seasonal restructuring, with winter‐dominant locations increasing from 29.63% (historical) to 45.68%, representing a change of +16.05 percentage points (pp). A nearly identical shift is observed in Uzbekistan (+16.67 pp), while Kazakhstan and Kyrgyzstan experience increases of +11.81 and +13.45 pp, respectively (see Figure 9; Figures S8–S11). These projected shifts highlight the increasing dominance of winter wind patterns under climate change scenarios, a pattern also reflected in studies like Zuo et al. [54], which reported increased seasonal variability and redistribution of wind energy availability across East Asia, especially under SSP5–8.5 [54].

This increase in winter dominance corresponds with sharp reductions in spring‐dominant locations across the same countries and time frame. For example, Tajikistan's spring share declines from 19.75% to 6.17% (–13.58 pp), while Uzbekistan drops from 27.78% to 13.89% (–13.89 pp) and Turkmenistan from 12.50% to 0.00% (–12.50 pp) (see Figures S8–S11). Declines are also notable in Kazakhstan (–7.64 pp) and Kyrgyzstan (–8.97 pp). These transitions are visually apparent in the season‐to‐season distribution figures, which show the dominant seasonal outcomes at each location over time (see Figure 9). While Zuo et al. [54] focus on China, they similarly observed substantial inter‐seasonal rebalancing of wind power contributions under SSP5–8.5, suggesting that changing atmospheric circulation patterns could be a common driver across the broader mid‐latitude region [54].

Although these changes are most dramatic under SSP5–8.5, a similar pattern exists under SSP2–4.5, albeit less severe. For instance, Uzbekistan and Tajikistan still exhibit increases in winter dominance of +16.67 and +16.05 pp, respectively, yet maintain greater portions of spring‐dominant areas compared to the higher‐emission scenario (see Figures S8–S11). Summer and fall dominance remain minimal in most contexts, with a few exceptions. Turkmenistan, under SSP5–8.5 (far‐future), shows a summer increase from 0% to 6.25%. Minor summer reductions are seen in Kazakhstan and Uzbekistan, the latter decreasing from 5.56% to 2.78% in the near future (see Figures S8–S11). This relative stability in seasonal dominance for some regions is consistent with findings by Meza‐Carreto et al. [55], who noted limited seasonal restructuring in certain inland parts of Mexico under future climate scenarios [55].

These regional and temporal changes are further supported by the seasonal distribution summaries, which aggregate the percentage of locations dominated by each season under different scenarios and periods. When considered alongside the location‐specific seasonal transitions, the overall picture confirms a substantial shift toward winter‐prevalent wind conditions, particularly in high‐elevation and continental regions. In summary, the transition toward winter wind regimes—already evident in some countries by the near‐future period, 2022–2051, has clear implications for long‐term wind resource planning. The moderate‐emission SSP2–4.5 scenario indicates that emissions mitigation can slow these transitions and preserve seasonal diversity. Adaptation strategies should focus on winter‐optimized turbine systems, seasonal storage technologies, and cross‐seasonal renewable integration to ensure resilient energy production in response to evolving seasonal wind regimes.

### 3.5 Seasonal Wind Speed Variability and Its Impact on Wind Energy Potential in CA

**FIGURE 10:**
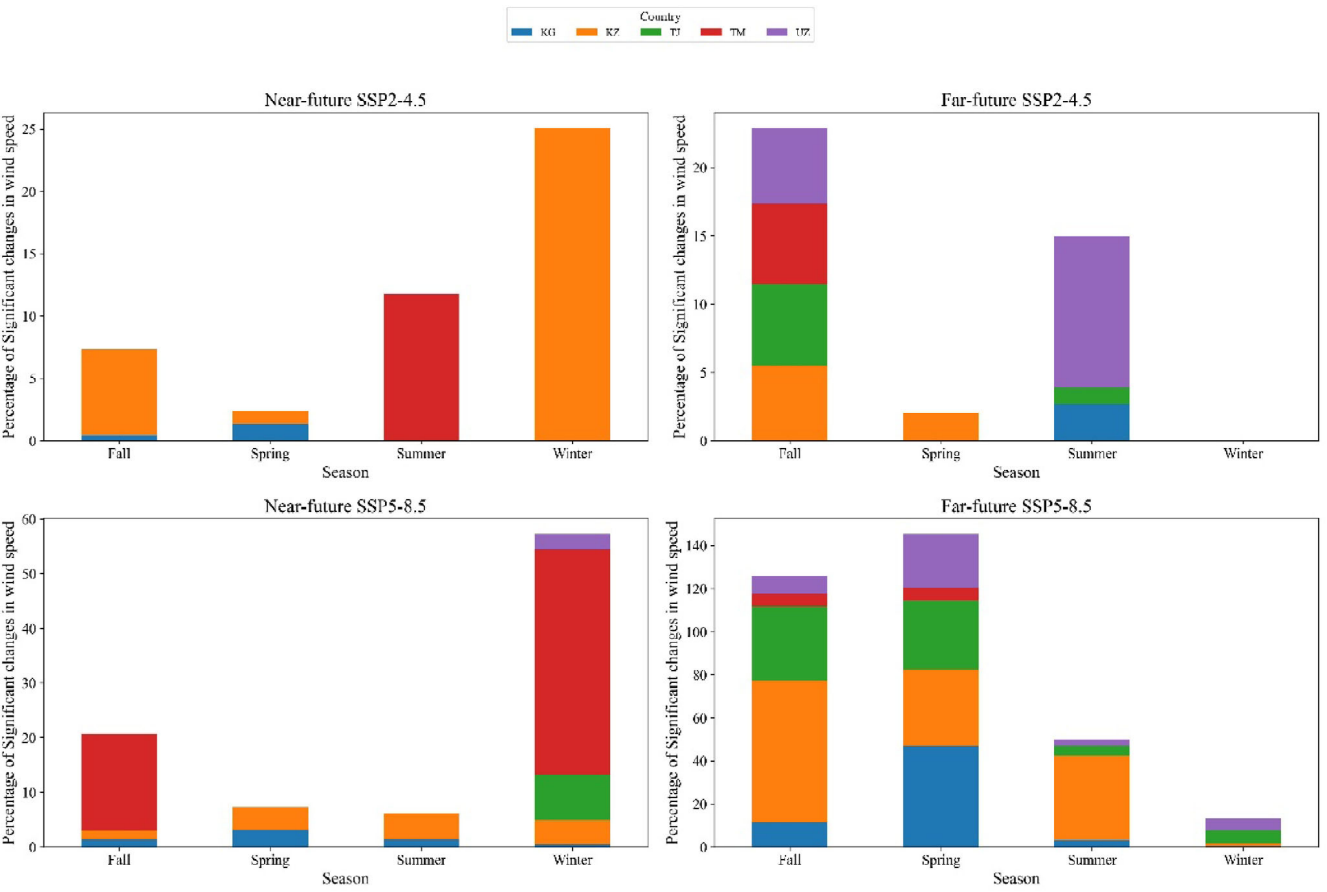
Comparative seasonal distribution of significant changes in wind speed across Central Asian countries under SSP2–4.5 and SSP5–8.5 scenarios for near‐future, 2022–2051, and far‐future, 2071–2100, periods.

Figure 10 shows the percentage of locations exhibiting statistically significant changes in seasonal wind speed, grouped by season (*x*‐axis) and disaggregated by country (stacked areas). The visualization reflects the contribution of each country to the overall seasonal wind signal in each scenario and time frame, with values representing deviations from historical baseline conditions rather than absolute wind speed values.

Our analysis under SSP2–4.5 and SSP5–8.5 for both the near‐future, 2022–2051, and far‐future, 2071–2100, reveals widespread seasonal wind speed variability across most regions, with a notable increase in the proportion of statistically significant seasonal deviations. Winter remains the most stable season, showing the lowest percentages of significant variability in Kazakhstan (1.37%), Kyrgyzstan (0.45%), and Tajikistan (5.95%) under SSP5–8.5 in the far‐future (see Figure 10), where winter occupies the smallest visual share, reinforcing its reliability under future climate conditions. By contrast, spring and fall demonstrate the highest levels of seasonal variability, especially under SSP5–8.5. In the far future, fall deviations are most prominent in Kazakhstan (65.64%), Tajikistan (34.52%), and Kyrgyzstan (11.61%), while spring deviations are notable in Kyrgyzstan (46.88%), Kazakhstan (35.40%), and Tajikistan (32.14%). This dominance is visually confirmed in the far‐future SSP5–8.5 (see Figure 10), where spring and fall comprise the majority of seasonal variation. These patterns suggest that transitional seasons may pose the greatest uncertainty in future wind energy reliability, underscoring the need for targeted seasonal planning. Kazakhstan emerges as the most sensitive to fall variability, followed by Tajikistan.

Summer variability is generally less frequent, though Kazakhstan (39.18%) and Kyrgyzstan (3.12%) show notable seasonal fluctuations under SSP5–8.5. Turkmenistan, which remains largely stable across scenarios, exhibits a brief period of summer variability (11.76%) under SSP2–4.5 in the near future. This transient behavior is visible in the near‐future SSP2–4.5 (see Figure 10), where Turkmenistan's summer signal briefly expands before disappearing in the far‐future, suggesting limited strategic relevance.

Near‐future scenarios reflect lower overall variability, with most seasonal deviations remaining below 5%, particularly under SSP2–4.5. This allows for higher confidence in mid‐term wind energy planning. However, far‐future projections, especially under SSP5–8.5, introduce substantially greater seasonal uncertainty—especially in spring and fall. Kazakhstan stands out with the highest levels of seasonal variability, while Turkmenistan and Uzbekistan maintain more stable profiles across timeframes.

These findings are strongly corroborated by recent literature. Miao et al. [50] and Zuo et al. [54] show that spring and fall are the most unstable seasons for wind generation across Asia, with winter consistently emerging as the most reliable. Moradian et al. [49] further confirm this seasonal risk profile using Copula and Weibull distribution modeling, while Meza‐Carreto and Romero‐Centeno [55] highlight the intra‐annual degradation of wind stability under SSP scenarios [54, 55]. These studies validate the Central Asian trends observed here and emphasize that seasonal instability—particularly in spring and fall—is a broad regional challenge, not limited to individual countries.

To address these evolving dynamics, energy planners should prioritize winter‐dominant strategies and avoid overdependence on spring or fall conditions. The results point to the necessity of hybrid system integration, grid flexibility, and energy storage solutions to buffer seasonal inconsistencies. As stronger emissions trajectories amplify variability, especially in transitional seasons, adaptive infrastructure and forward‐looking energy models will be crucial for maintaining wind energy reliability across CA.

### 3.6 Weibull‐Based Analysis of Characteristic Wind Speed Across CA

**FIGURE 11:**
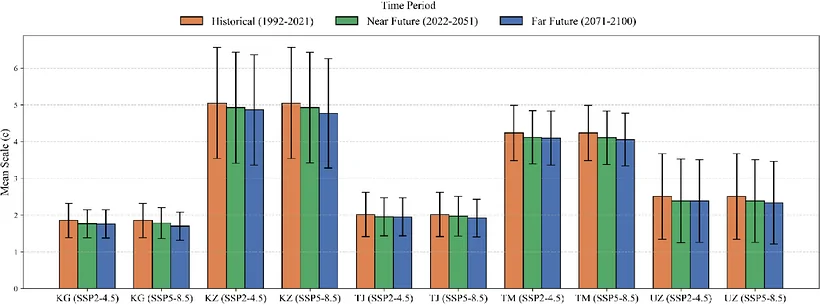
Weibull scale statistics across Central Asian countries under SSP2–4.5 and SSP5–8.5 scenarios for historical, 1992–2021, near‐future, 2022–2051, and far‐future, 2071–2100, periods.

Figure 11 shows the Weibull scale parameter (*c*), representing average wind speed and energy potential. Bars indicate the mean values across grid locations, and error bars represent standard deviations, reflecting statistical variability. This study evaluates the wind energy potential of Kazakhstan, Kyrgyzstan, Turkmenistan, Uzbekistan, and Tajikistan over three time periods—historical, 1992–2021, near‐future, 2022–2051, and far‐future, 2071–2100, under SSP2–4.5 and SSP5–8.5. The analysis focused on the Weibull scale parameter as an indicator of characteristic wind speed and associated energy potential across different climate scenarios.

Kazakhstan continues to demonstrate high wind energy potential, with a historical Weibull scale mean of 5.05. Future projections reveal gradual declines in scale values under both SSP2–4.5 and SSP5–8.5. Wind speeds decline gradually over time, with the scale parameter dropping from 5.02 to 4.98 (near‐future) and from 4.86 to 4.77 (far‐future) under SSP5–8.5 (see Figure 11).

These results highlight the gradual weakening of characteristic wind speeds across most Central Asian countries, particularly under SSP5–8.5. Kyrgyzstan presents a contrasting profile—moderate baseline performance but exceptional stability. The scale parameter remains largely unchanged, slightly rising from 1.76 to 1.78 and then declining to 1.69. This indicates low sensitivity to climate‐driven changes, making Kyrgyzstan one of the most stable environments for long‐term wind energy investment. Turkmenistan displays a unique profile with relatively low sensitivity and stable performance across scenarios. Historical and projected scale values remain relatively unchanged, confirming that Turkmenistan experiences minimal scenario‐driven variation and supports consistent investment potential.

Uzbekistan is marked by moderate wind performance but higher volatility. Scale values improve slightly (3.12 → 3.19), suggesting stronger winds. However, in the far future, shape diverges further (10.14 → 9.85), and scale drops (3.25 → 3.12), confirming long‐term instability (see Figure 11). Tajikistan is the most stable among all countries. Scale remains nearly constant, dipping slightly from 1.73 to 1.69 (see Figure 11). This stability reinforces Tajikistan's classification as a region with minimal climate‐driven variability. These findings confirm that Kyrgyzstan and Tajikistan offer the most stable environments for wind energy development in CA, particularly under SSP5–8.5. Kazakhstan and Uzbekistan, by contrast, present significant trade‐offs between strong wind speeds and long‐term variability.

Turkmenistan sits in the middle, with notable temporal shifts but low scenario sensitivity. Overall, our analysis highlights how stronger emissions scenarios amplify wind variability, particularly in countries already exposed to changes in characteristic wind speeds. This underscores the need for adaptive infrastructure, hybrid renewable systems, and advanced forecasting—especially in Kazakhstan and Uzbekistan—to ensure long‐term energy reliability. Meanwhile, stable environments like Kyrgyzstan and Tajikistan can support more conventional wind deployment with minimal climate‐induced disruptions.

This trend of declining scale values is consistent with recent literature modeling Weibull scale evolution under climate change. Kashyap (2024) and Miao et al. [50] both report significant decreases in characteristic wind speed across global and Northern Hemisphere sites under SSP5–8.5. Moradian et al. [49], using Copula‐based distribution fitting, confirm similar declines and suggest zonal planning for future installations [49]. Barkanov et al. [48] likewise observed a flattening of wind distributions in European offshore zones, reflecting reduced high‐wind occurrences. These findings validate the projections observed in CA, reinforcing the robustness of using Weibull parameters to assess wind energy reliability under future climate scenarios [48].

The significant variability and complexity of wind energy potential across CA, as identified in Sections 3.1–3.4, necessitate a robust and comprehensive ranking methodology that accounts for both short‐term seasonal fluctuations and long‐term wind resource stability. The analyses of seasonal wind speed classifications (Section 3.1), seasonal shifts in wind speeds (Section 3.2), seasonal wind speed variations (Section 3.3), and Weibull‐based wind speed analysis (Section 3.4) demonstrate that a ranking system based solely on mean wind speeds would be inadequate in assessing long‐term wind energy feasibility.

To address these complexities, the ranking framework employed in this study incorporates multiple key factors beyond mere wind speed averages. The methodology integrates Weibull‐based wind speed characterization (capturing the representative or characteristic wind speed at each site), exceedance probabilities (assessing the frequency of wind speeds within optimal operational thresholds for turbines), and interannual wind variability trends (evaluating fluctuations in wind speeds over time). This ensures that locations exhibiting seasonal wind speed declines may still be ranked favorably if they demonstrate strong overall wind resource characteristics, while regions experiencing increased anomalies under SSP5–8.5 may undergo ranking fluctuations due to shifting exceedance probabilities.

This approach ensures that the ranking methodology remains scientifically rigorous, adaptable to changing wind conditions, and capable of capturing both short‐term seasonal variations and long‐term stability trends. As a result, the rankings presented in this study provide a balanced and holistic assessment of wind energy potential across CA, ensuring that site selection reflects not only wind speed magnitudes but also the reliability and operational feasibility of wind resources over time.

### 3.7 Long‐Term Wind Energy Ranking Trends Under SSP2–4.5 and SSP5–8.5 Scenarios in CA

Building on the statistical characterization of wind speed and variability presented in previous sections, this section evaluates how these changes translate into site‐level wind energy rankings. In CA, the analysis of wind energy rankings across the historical period, 1992–2021, near‐future, 2022–2051, and far‐future, 2071–2100, under SSP2–4.5 and SSP5–8.5 reveals distinct spatial and temporal trends in wind resource suitability. Across both scenarios, a considerable number of locations experience meaningful shifts in their relative wind energy potential, with some areas consistently improving in rank while others decline. Overall, the results indicate that wind energy suitability across CA is undergoing widespread but spatially heterogeneous change, with a general tendency toward declining rankings under both scenarios, particularly under SSP5–8.5. This indicates that future wind energy development in CA will become increasingly location‐dependent, requiring site‐specific evaluation rather than reliance on historical suitability patterns.

Wind energy suitability was evaluated independently for each location, time period, and scenario using a ranking system, where a lower rank corresponds to higher wind potential. This approach enables direct comparison across scenarios and timeframes, offering insights into how wind suitability evolves under different climate trajectories. Near‐future rankings are particularly relevant for short‐ to mid‐term infrastructure planning, while far‐future projections support long‐term strategy development and resilience building under climate change. Unless otherwise stated, the values discussed in this section refer either to absolute ranking positions or to rank changes relative to the historical baseline. Positive rank changes indicate worsening suitability (movement toward a higher numerical rank), whereas negative rank changes indicate improved suitability.

The top‐ranked locations across CA under different scenarios and time periods are provided in Table S2. Under SSP2–4.5, only 22.04% of locations improve in the near future compared to their historical rankings, while 74.32% experience a decline, suggesting a more widespread reduction in wind energy potential than previously anticipated. Similar trends emerge under SSP5–8.5, with 22.19% of locations improving and 75.23% declining, a pattern broadly consistent with SSP2–4.5 but reflecting slightly greater deterioration in wind rankings. These results suggest that declining suitability is not scenario‐specific but represents a consistent regional trend across CA.

**FIGURE 12:**
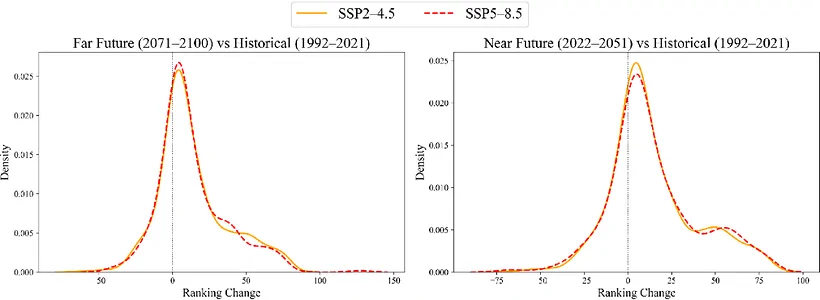
Density distributions of wind energy suitability ranking changes for all evaluated locations across Central Asia. The plots compare future periods—near‐future, 2022–2051, and far‐future, 2071–2100, against the historical baseline, 1992–2021, under two emission scenarios: SSP2–4.5 and SSP5–8.5.

**FIGURE 13:**
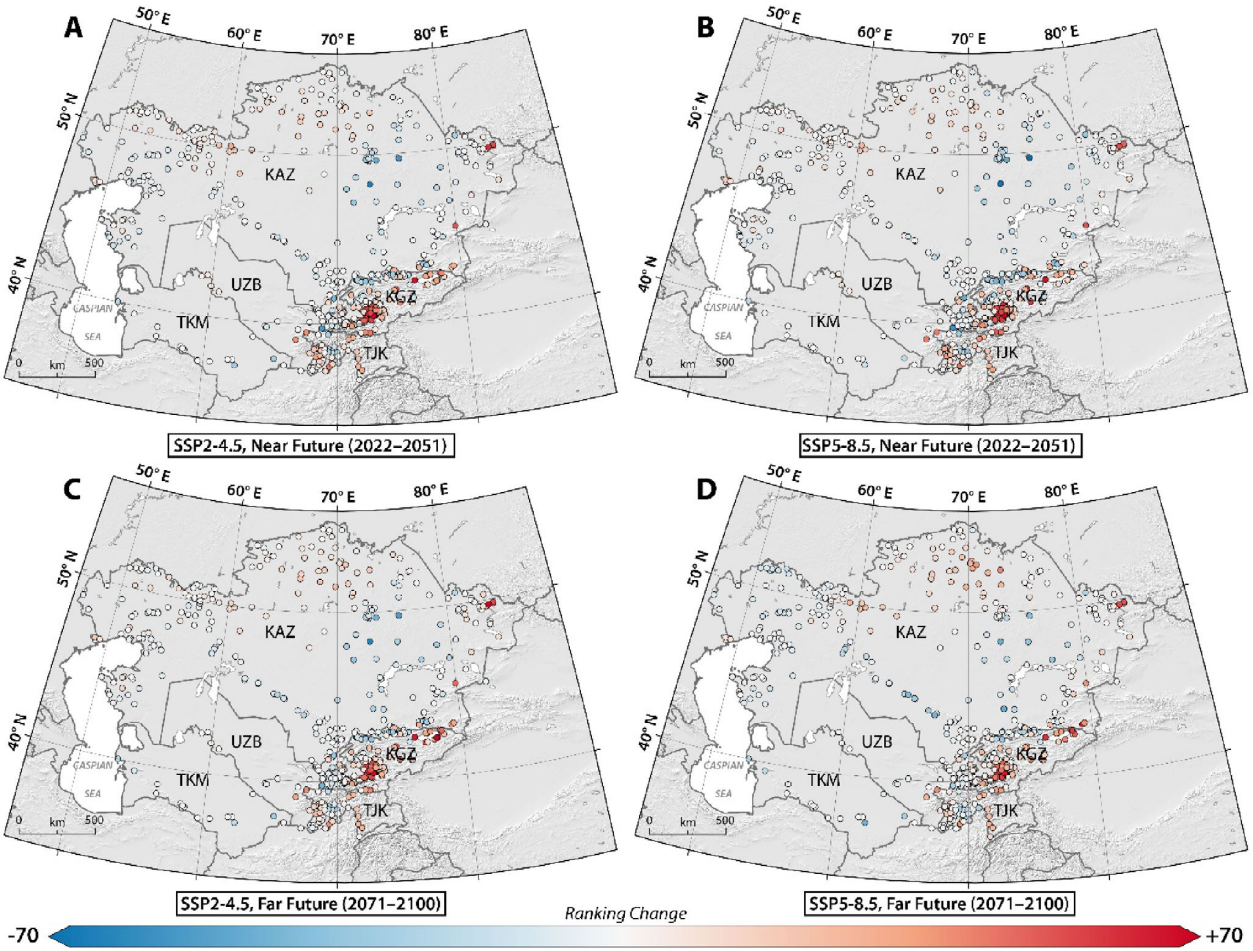
Geospatial distribution of wind energy suitability ranking changes under SSP2–4.5 and SSP5–8.5 for the near‐future, 2022–2051, top, and far‐future, 2071–2100, bottom, relative to the historical baseline, 1992–2021. Each point represents one of the ERA5 grid locations used in the analysis across CA, with colors indicating the magnitude and direction of ranking change. Warm colors (red/yellow) denote worsening ranks (i.e., decreasing wind potential), while cool colors (blue) indicate improved suitability.

To further illustrate these scenario‐driven changes, geospatial plots of ranking change and kernel density estimations (KDEs) were developed (see Figure 12). The geospatial maps visualize the regional concentration of ranking improvements and deteriorations (see Figure 13), while the KDE plots provide a statistical overview of the distribution and intensity of rank changes across all sites.

In the near future, both SSP2–4.5 and SSP5–8.5 exhibit unimodal, symmetric distributions centered close to zero, indicating that most sites undergo only moderate changes in rank relative to the historical baseline. However, under SSP5–8.5, the curve becomes noticeably broader with a heavier rightward tail, suggesting that a growing subset of locations is beginning to experience more substantial deterioration in suitability. In the far future, divergence becomes clearer. The SSP2–4.5 distribution remains compact, with 75% of sites falling within ±50 rank changes. In contrast, SSP5–8.5 shows a flatter and more right‐skewed distribution, with ranking changes ranging from improvements of over –40 to declines exceeding +120. This implies that under a high‐emissions scenario, some locations could lose up to 120 places in suitability rankings compared to their historical performance, a statistically significant signal of long‐term climate impact. These distributional patterns indicate that while most locations experience moderate ranking shifts, a subset of sites undergoes substantial deterioration, particularly under SSP5–8.5. This highlights an increasing concentration of wind energy potential in fewer locations, while a growing number of sites become less viable.

The geospatial ranking change maps complement these findings by visualizing the spatial concentration of rank change (see Figure 13). Under SSP2–4.5, ranking changes are mild and more evenly distributed across CA, with slight improvements observed in parts of western Kazakhstan and northern Turkmenistan. Under SSP5–8.5, particularly in the far‐future, regions such as eastern Kazakhstan, central Kyrgyzstan, and southern Tajikistan display clusters of substantial rank deterioration. Conversely, isolated pockets of resilience—such as locations in southwestern Kazakhstan—still show improvement. These spatial patterns confirm that wind suitability under high‐emission futures is not only more variable but also more geographically uneven, highlighting the need for fine‐scale spatial prioritization in infrastructure planning. This suggests that future wind energy investments should increasingly focus on localized high‐performing zones rather than broad regional deployment strategies.

In terms of location‐specific trends, some areas demonstrate exceptional ranking improvements across historical, near‐future, and far‐future periods. Aqadyr, Kazakhstan (115 → 52 → 55 under SSP2–4.5 and 115 → 50 → 73 under SSP5–8.5) shows a strong and consistent improvement, reinforcing its potential as a promising wind energy development site in both scenarios. Koktal, Kazakhstan (92 → 48 → 53 under SSP2–4.5 and 92 → 49 → 76 under SSP5–8.5) also shows meaningful improvement, illustrating its potential as a stable and strengthening wind energy site over time. Prigorodnoye (141 → 88 → 92 under SSP2–4.5 and 141 → 72 → 107 under SSP5–8.5) demonstrates a similar upward trend, highlighting its growing suitability for future wind energy development. Likewise, Shahriston, Tajikistan (415 → 366 → 377 under SSP2–4.5 and 415 → 361 → 393 under SSP5–8.5) indicates promising potential despite starting from a lower baseline. These examples demonstrate that ranking improvements are not uniformly distributed but are concentrated in specific locations, primarily in Kazakhstan, suggesting emerging regional advantages under future climate conditions.

Conversely, several locations experience substantial declines in their wind energy rankings over time. Saruu, Kyrgyzstan (313 → 355 → 391 under SSP2–4.5 and 313 → 331 → 440 under SSP5–8.5) indicates a significant reduction in wind energy suitability. Pervorossiyskoye (350 → 418 → 428 under SSP2–4.5 and 350 → 411 → 412 under SSP5–8.5) also shows a pronounced decline, while Nariman (365 → 442 → 440 under SSP2–4.5 and 365 → 444 → 438 under SSP5–8.5) and Osh (365 → 442 → 440 under SSP2–4.5 and 365 → 444 → 438 under SSP5–8.5) both drop from the mid‐300s historically to over 440 in the far‐future. These deteriorating trends highlight the importance of reevaluating long‐term wind energy potential in areas facing increasing atmospheric instability or regional circulation shifts. These declines indicate that certain regions—particularly in Kyrgyzstan and Tajikistan—may face increasing limitations for long‐term wind energy development.

In contrast to regions experiencing major shifts, some locations maintain exceptional stability in wind energy potential across both moderate and high‐emission scenarios. To identify these stable performers, only locations with a historical rank in the top 50 (i.e., strong baseline wind potential) were considered. From this subset, locations were included if their rankings changed by no more than ±10 ranks between the historical and far‐future periods under both SSP2–4.5 and SSP5–8.5 scenarios. This filtering approach ensures that selected sites demonstrate both high suitability and low variability, making them ideal candidates for low‐risk, long‐term energy infrastructure. Based on this analysis, Akshukyr (2 → 2 → 2 under SSP2–4.5 and 2 → 2 → 2 under SSP5–8.5) stands out as a remarkably consistent performer. Aqqyztoghay (19 → 19 → 19 under SSP2–4.5 and 19 → 19 → 19 under SSP5–8.5) also exhibits perfect stability. Akbakay (18 → 18 → 12 under SSP2–4.5 and 18 → 13 → 12 under SSP5–8.5) shows minor improvement. Akkystau (14 → 13 → 17 under SSP2–4.5 and 14 → 15 → 18 under SSP5–8.5) and Akshimrau (24 → 26 → 27 under SSP2–4.5 and 24 → 29 → 25 under SSP5–8.5) remain in the top 30 with only slight variation, suggesting a high degree of atmospheric stability favorable to wind development. Importantly, none of the top 50 locations showed any significant decline (i.e., greater than 100‐rank drop) in either emission scenario, reinforcing their status as highly reliable and resilient wind energy sites.

While near‐future trends exhibit some regional consistency, our analysis shows that over 74% of locations experience a decline in wind energy ranking from historical to near‐future periods under both SSP2–4.5 and SSP5–8.5. This suggests that even in the near‐term, atmospheric variability and evolving circulation patterns are likely to affect site suitability more than previously assumed. Far‐future projections introduce even greater uncertainty due to increasing variability in modeled wind patterns, compounded by inherent limitations in CMIP6 climate model outputs [56, 57]. Long‐term projections of atmospheric circulation changes—particularly in complex systems such as jet streams, Hadley cells, regional pressure gradients, and stratospheric circulation—exhibit growing uncertainty due to model resolution constraints, inter‐model discrepancies, and the complexity of stratosphere–troposphere interactions [58]. Taken together, these findings indicate that future wind energy development in CA will likely become more spatially selective, with a smaller number of high‐performing and stable locations driving investment potential. This underscores the importance of prioritizing resilient sites while avoiding long‐term commitments in areas exhibiting strong ranking deterioration.

Although wind rankings are primarily driven by exceedance probabilities and Weibull‐based wind speed characterization, rather than seasonal anomalies alone, increasing wind variability under SSP5–8.5 could still introduce localized ranking deviations not fully captured in long‐term trend projections. Given that both scenarios show similar patterns in the direction of change, investment decisions in the near future may still be made with moderate confidence, particularly for locations with stable historical performance and minimal variability. However, sites with known high seasonal wind anomalies, especially in spring and fall, should be approached with added caution.

Since emissions scenarios do not appear to drastically shift regional rankings in the near‐term, investment strategies should be guided more by observed wind behavior and atmospheric consistency, rather than long‐term emissions assumptions alone. These findings emphasize the importance of a data‐driven, risk‐aware approach to wind energy planning, incorporating climate projections to strategically deploy infrastructure in regions with stable or improving wind potential. Additionally, hybrid renewable systems and flexible grid solutions should be prioritized in areas with fluctuating wind conditions to enhance energy security and resilience in a changing climate [59].

### 3.8 Ranking Changes Analysis Across Central Asian Countries

**FIGURE 14:**
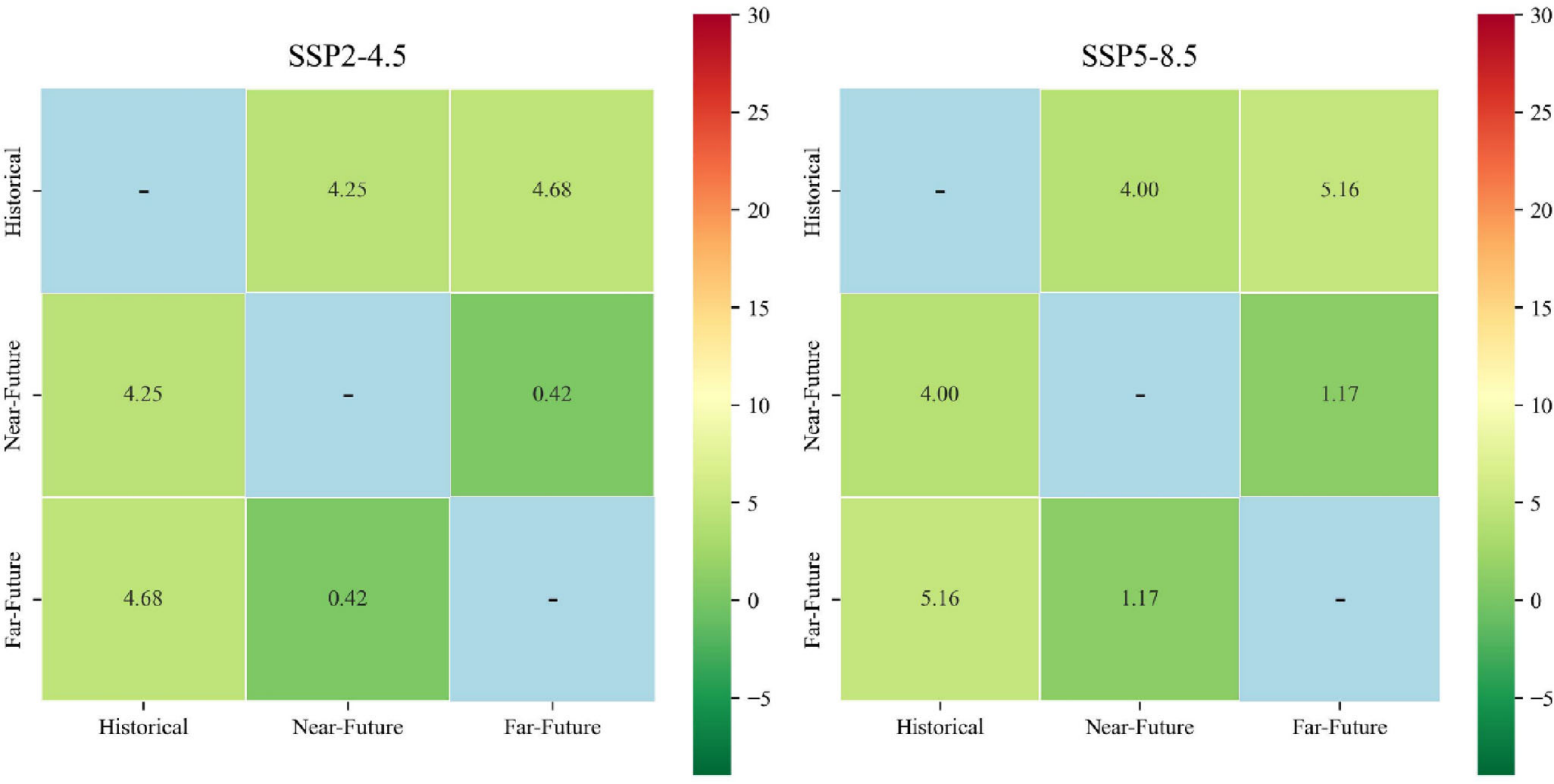
Mean rank changes (%) in location‐based wind energy ranking scores in Kazakhstan relative to the historical baseline, under SSP2–4.5 and SSP5–8.5 scenarios.

Figure 14 and Figures S12–S15 show the changes in wind resource rankings across Central Asian countries, based on location‐specific suitability scores. These heatmaps present the mean absolute difference in wind ranking scores between the historical baseline, 1992–2021, and two projected periods: the near‐future, 2022–2051, and the far‐future, 2071–2100, under both SSP2–4.5 and SSP5–8.5 scenarios. Thus, the values in this section refer to average rank changes, not absolute wind speed values; when country‐average ranking positions are discussed, they are stated explicitly.

Overall, the country‐level analysis reveals contrasting trajectories in wind energy suitability, with some countries exhibiting strong stability and others experiencing substantial degradation in ranking performance.

Kazakhstan emerged as the most favorable country, with a country‐average ranking position changing only slightly from 143 historically to 147 in the far‐future under SSP2–4.5 and to 148 under SSP5–8.5, corresponding to mean rank changes of +4.45 and +5.04, respectively. This long‐term consistency is further illustrated in the heatmaps for Kazakhstan (see Figure 12), which show very limited ranking shifts over time, particularly between the near‐ and far‐future periods, where changes remain below 1.2 rank units in both scenarios. This indicates that Kazakhstan maintains relatively stable wind energy suitability over time, making it a reliable candidate for long‐term investment despite minor ranking deterioration.

In contrast, Kyrgyzstan experienced the most significant decline, with average rankings worsening by +27 (SSP2–4.5) and +28 (SSP5–8.5) in the near‐future and by +27 and +26 in the far‐future; however, the heatmap analysis (see Figures S1–S15) shows that most of this degradation occurs early, between the historical and near‐future periods, while the difference between near‐ and far‐future ranks is minimal (–0.45 to –1.10), indicating a relative stabilization of wind potential after the initial drop. This suggests that Kyrgyzstan undergoes an early structural decline in wind suitability, after which conditions stabilize at a lower performance level, indicating reduced long‐term competitiveness for wind energy development.

Uzbekistan followed similar trends, with ranking deterioration of +25 and +21 under SSP2–4.5 and SSP5–8.5, respectively, by the far‐future. The heatmaps (see Figure S12) indicate that this decline primarily occurs between the historical and near‐future periods (+29 and +25), with a modest improvement between near‐ and far‐future projections (–3.99 and –4.11), suggesting that wind potential in Uzbekistan may partially stabilize in the second half of the century. This pattern indicates that Uzbekistan is highly sensitive to near‐term changes but may experience partial recovery or stabilization over longer timescales, highlighting the importance of adaptive planning strategies.

Tajikistan's ranking shift was more moderate, with changes of +9.92 under SSP2–4.5 and +7.31 under SSP5–8.5 from historical to the far‐future (see Figure S13). However, in Tajikistan, most of the ranking loss occurs in the near‐future (+12 and +13, respectively), followed by a partial recovery in the far‐future (–2.23 and –5.81), particularly under SSP5–8.5, suggesting that wind potential may stabilize or even slightly improve after mid‐century (see Figure S13). This indicates a transitional response, where initial deterioration is followed by partial recovery, suggesting moderate resilience compared to other declining regions.

Turkmenistan exhibited minor improvements in the far‐future under SSP5–8.5 (–4), making it a potential emerging zone of interest. This trend is reinforced by the heatmaps (see Figure S14), which show virtually no degradation under SSP2–4.5 and a modest but consistent improvement under SSP5–8.5, particularly from the near‐ to far‐future period (–6), indicating that long‐term wind potential may strengthen in a high‐emissions scenario. This suggests that Turkmenistan may represent a relatively stable or even improving wind energy region under future climate conditions, particularly under higher emission scenarios.

To identify the most investment‐ready sites, we applied a refined classification to isolate stable high‐performing locations that met three quantitative thresholds: (1) a historical rank ≤100, (2) a change from historical to the far‐future of no more than ±10 ranks under SSP2–4.5, and (3) a similar ±10 variation under SSP5–8.5. This ensured the selection of sites with strong baseline wind conditions and resilience to long‐term climate shifts. Figure 12 shows the percentage of sites in each country falling into five predefined performance tiers, where Tier 1 represents the most favorable wind conditions for energy generation and Tier 5 the least. All qualifying sites were located in Kazakhstan and included Fort‐Shevchenko (1 → 1 → 1), Bautino (1 → 1 → 1), Akshukyr (2 → 2 → 2), Yerkinkala (3 → 4 → 6), and Balykshi (3 → 4 → 6), all of which exhibited near‐perfect stability across both scenarios and timeframes.

**FIGURE 15:**
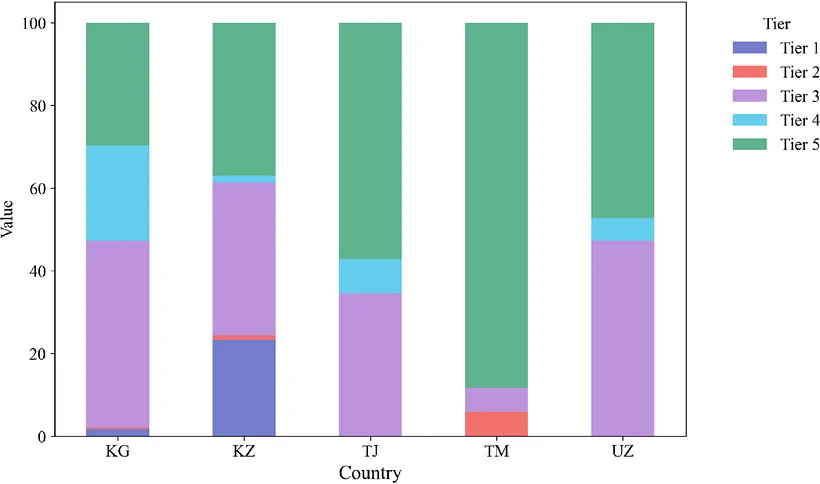
Distribution of wind energy suitability tiers across Central Asian countries based on historical wind conditions.

The tier distribution varies significantly by country (see Figure 15), with Kazakhstan accounting for 23.4% of its sites in Tier 1, the highest share of stable, high‐performing locations in the region. Kyrgyzstan exhibits the greatest proportion of Tier 4 sites (23%), indicating widespread long‐term decline, while Turkmenistan and Tajikistan show exceptionally high proportions of Tier 5 sites (88.2% and 57.1%, respectively), suggesting strategic ambiguity and mixed performance outcomes. Uzbekistan is almost evenly split between Tier 3 and Tier 5 (47.2% each), reflecting strong scenario sensitivity and moderate reliability.

These contrasts highlight a clear regional differentiation, where Kazakhstan stands out as the most stable and investment‐ready country, while Kyrgyzstan, Uzbekistan, and Tajikistan require more cautious and adaptive planning approaches due to higher variability and decline. These findings reinforce the importance of tailoring investment strategies to country‐specific wind energy dynamics and emissions pathway responsiveness.

Tier 1 sites, such as Fort‐Shevchenko or Akshukyr, are prime candidates for immediate large‐scale deployment. Tier 2 sites—such as Aqadyr (115 → 55 → 73) and Koktal (92 → 53 → 76)—offer high improvement potential and warrant prioritized monitoring. Tier 3 sites, including many in Uzbekistan and Turkmenistan, highlight the importance of emissions‐sensitive modeling in infrastructure planning. Tier 4, heavily represented in Kyrgyzstan, consists of locations in long‐term decline and better suited for backup or complementary energy solutions. The inclusion of Tier 5 recognizes the strategic ambiguity of many sites and underscores the value of continuous monitoring and localized assessment. These findings emphasize the necessity of integrating emissions scenario uncertainty, long‐term projections, and temporal trends into wind energy development frameworks. The distribution of sites across the five‐tier framework reveals important insights about the long‐term outlook for wind energy development in CA. Only 11.1% of locations fall into Tier 1, representing highly stable and investment‐ready zones concentrated primarily in Kazakhstan. In contrast, nearly 40% of sites (Tier 3) are scenario‐sensitive, suggesting widespread emissions‐dependent variability that must be accounted for through adaptive planning and hybrid infrastructure. Tier 4 sites—comprising 10% of the region—show sustained decline and require cautious evaluation, while nearly 39% of locations fall into Tier 5, indicating moderate or mixed behavior and may require case‐by‐case evaluation depending on local factors, grid integration opportunities, or co‐located energy systems.

Taken together, these results indicate that CA's future wind energy landscape will be shaped by a combination of high‐confidence anchor sites and a broader set of context‐dependent locations. Wind energy development is therefore becoming increasingly location‐dependent, with clear contrasts emerging across countries and scenarios, reinforcing the need for targeted, data‐driven, and region‐specific planning strategies.

A comparative summary of ranking trends, stability classifications, drivers of variability, and strategic planning recommendations for each country is presented in Table 4.

**TABLE 4:** Comparison of wind energy ranking trends and strategic planning needs across Central Asian countries.

Country	Ranking trend over time	Stability pattern (tier distribution)	Drivers of variability	Energy planning strategy
Kazakhstan	Minor average rank shift (+5 to +5); highly stable over time and across scenarios	Tier 1 (23.4%), Tier 3 (36.9%), Tier 5 (36.9%)	Minor seasonal anomalies under SSP5–8.5 in site selections	Prioritize early investment in high‐performing near‐future sites with low scenario sensitivity
Turkmenistan	Modest long‐term improvement under SSP5–8.5; scenario influence less predictable	Tier 2 (5.9%), Tier 3 (5.9%), Tier 5 (88.2%)	Site‐level exceedance variability and microclimate effects	Target high‐performing sites selectively; re‐evaluation is essential before long‐term deployment
Uzbekistan	Sharp early degradation (+25 SSP2–4.5, +21 SSP5–8.5), slight stabilization after 2051	Tier 3 (47.2%), Tier 5 (47.2%)	Emissions pathway sensitivity and seasonal wind variability	Use site‐specific, adaptive planning; prioritize semi‐stable performers and monitor scenario influence
Tajikistan	Modest rank changes (+7 to +10) with partial recovery in the far‐future	Tier 3 (34.5%), Tier 5 (57.1%)	Local terrain and regional circulation variability are not strongly driven by the emissions scenario	Phase investments with long‐term reassessment checkpoints; avoid early full‐scale deployment
Kyrgyzstan	Major early deterioration (+27 in the near‐future), followed by slight far‐future stabilization	Tier 3 (45.1%), Tier 4 (23%), Tier 5 (29.7%)	Scenario sensitivity under SSP5–8.5 and complex terrain dynamics	Avoid wind‐exclusive infrastructure; consider hybrid systems with staggered deployment and ongoing monitoring

*Note*: The “Drivers of Variability” column summarizes key factors influencing wind energy ranking shifts within each country. These are derived from the Tier‐based classification system rather than statistical correlations. Specifically:

• Countries with a high proportion of Tier 3 sites are classified as scenario‐sensitive, reflecting dependence on emissions pathways (i.e., differences between SSP2–4.5 and SSP5–8.5).

• Countries dominated by Tier 5 reflect mixed or ambiguous behavior, where site performance does not follow a consistent pattern and may be influenced by local microclimates, exceedance probabilities, or model uncertainty.

• When both Tier 3 and Tier 5 are present in near‐equal proportions, the driver is noted as balanced or dual.

• Tier 4 presence points to declining site performance over time, often driven by longer‐term weakening of wind potential.

These assignments are supported by site‐level trend analysis and heatmap observations, which illustrate spatial and temporal variability in ranking changes under both climate scenarios.

### 3.9 Cross‐Method Synthesis of Wind Energy Potential in CA

Across the multiple analytical approaches applied in this study—including seasonal trend analysis, wind speed categorization, variability assessment, Weibull‐based characterization, and ranking‐based evaluation—a set of consistent and complementary patterns emerges. Kazakhstan is identified as the most stable and investment‐ready region across all methods, maintaining relatively strong wind conditions, limited variability, and a high proportion of Tier 1 sites. In contrast, Kyrgyzstan shows a consistent decline in suitability, with strong deterioration in ranking‐based metrics despite moderate stability in some wind characteristics. Southern countries, particularly Turkmenistan and Tajikistan, show mixed or low‐performance signals across methods, reflecting higher uncertainty and spatial heterogeneity.

A key area of agreement across methods is the increasing influence of climate change on wind resource reliability. Under SSP5–8.5, all methods consistently indicate amplified variability, declining wind speeds, and increasing instability, particularly during transitional seasons (spring and fall). Winter emerges as the most stable season across both variability and ranking analyses, reinforcing its importance for future energy planning. Together, these results indicate that climate change not only reduces wind energy potential in several regions but also increases uncertainty in wind resource availability.

At the same time, methodological differences provide complementary insights into wind energy dynamics. While Weibull‐based metrics capture changes in average wind speed and distribution characteristics, ranking and tier‐based approaches reveal the combined effects of variability, exceedance probabilities, and long‐term stability. As a result, some locations with moderate wind speeds may still rank poorly due to high variability or declining reliability, while others maintain strong rankings despite modest changes in mean wind conditions.

Overall, these results demonstrate that no single metric is sufficient to fully capture wind energy suitability under changing climate conditions. Instead, an integrated framework—combining statistical, seasonal, and probabilistic analyses—offers a more robust and reliable basis for long‐term wind energy planning.

### 3.10 Methodological Comparison and Limitations

Table 5 presents a comparative analysis of our wind energy ranking methodology and traditional GIS‐based multi‐criteria decision‐making (MCDM) approaches such as AHP, TOPSIS, ELECTRE, and Fuzzy Logic. The comparison highlights key methodological differences and their implications for long‐term wind energy planning under climate change.

**TABLE 5:** Comparison of wind energy ranking methodologies: Traditional GIS‐MCDM approaches versus climate‐responsive future projections.

Criteria	Our method (future projections under SSP2–4.5 and SSP5–8.5)	Traditional wind farm ranking methods	Refs.
Temporal scope	Projects wind energy rankings for near‐future, 2022–2051, and far‐future, 2071–2100, under changing climate conditions	Mostly static or based on historical wind data with no climate projections	[60, 61]
Wind speed estimation	Uses Weibull‐based wind speed characterization for each season to model future wind trends	Often uses historical wind speed averages or direct wind power density values	[61, 62]
Climate change impact	Directly integrates climate model projections (future wind trends under different SSPs)	Assumes that past wind speed patterns remain stable (not always valid under climate change)	[60, 63]
Variability analysis	Computes wind variability trends over time, allowing ranking to favor stable locations	Some studies consider inter‐annual variability, but most do not include long‐term change trends	[64, 65]
Exceedance probabilities	Evaluates wind speed exceedance at critical operational thresholds (8–11, 12–24 m/s, etc.) to assess wind farm reliability	Some studies incorporate exceedance analysis, but it is not always central to ranking	[64, 65]
Ranking approach	Uses time‐weighted ranking adjustments to prioritize recent conditions and capture wind resource shifts over decades	Often uses multi‐criteria decision‐making (MCDM) approaches like TOPSIS, AHP, ELECTRE, which are typically not time‐sensitive	[61, 66]
Computational complexity	Uses probability distribution fitting (Weibull) and multi‐time‐period weighted ranking, making it more computationally intensive	GIS‐MCDM methods are computationally efficient, but less adaptable to long‐term projections	[66, 67]
Site selection outcome	Provides a ranking that evolves over time, enabling decision‐makers to adjust investment strategies for future wind conditions	Provides a single static ranking, which may not remain valid under future climate scenarios	[60, 61]

The selection of optimal wind energy sites requires integrating resource availability, stability, and long‐term viability. Traditional wind energy site selection approaches, such as GIS‐based multi‐criteria decision‐making (MCDM) methods (e.g., AHP, TOPSIS, ELECTRE, Fuzzy AHP), have been widely used due to their computational efficiency and ability to incorporate diverse site selection criteria [65, 66]. However, these methods rely primarily on historical wind data, assuming that past wind conditions remain stable, which may not be valid under a changing climate [60]. In contrast, the proposed climate‐adaptive ranking framework integrates long‐term wind projections under SSP2–4.5 and SSP5–8.5, enabling a dynamic, forward‐looking assessment of wind energy suitability.

A key distinction is the explicit integration of climate model projections to capture evolving wind patterns under different emissions pathways. Unlike traditional methods, which provide a single static ranking, the method generates time‐evolving rankings across historical, near‐future, and far‐future periods. This is achieved by computing Weibull probability distributions for wind speeds, allowing a probabilistic assessment of wind energy potential across seasons [61, 62]. Additionally, wind variability trends are incorporated to prioritize stable locations and reduce investment risk. Another advantage of our method is its explicit consideration of wind speed exceedance probabilities, particularly for critical operational ranges. This ensures that site selection reflects not only mean wind conditions but also operational reliability. Traditional methods often rely on mean wind speed values alone, potentially overlooking seasonal and interannual variability [64].

Time‐weighted ranking adjustments further ensure that rankings reflect recent and projected wind conditions. This results in a climate‐informed, site‐specific ranking system adapted to long‐term variability. Sites are categorized into performance tiers based on their historical strength, temporal ranking stability, emissions pathway sensitivity, and directional trends—allowing clear differentiation between stable, improving, and declining sites.

Despite these advantages, several limitations should be acknowledged. The reliance on climate model projections introduces uncertainties related to model accuracy, particularly in regions where wind dynamics are influenced by complex local atmospheric interactions [60]. Although a multi‐model CMIP6 ensemble was used to reduce bias, the analysis focuses on ensemble means and does not explicitly quantify inter‐model spread or probabilistic uncertainty [53].

In addition, the study uses ERA5 reanalysis data as the observational reference for bias correction. While ERA5 provides high‐quality and spatially consistent data, discrepancies may exist relative to in situ measurements, particularly in complex terrain or data‐sparse regions.

Additionally, the computational complexity of our method—requiring Weibull‐based wind speed characterization, exceedance probability calculations, tier‐based classification, and multi‐time‐period ranking adjustments—is significantly higher than traditional GIS‐MCDM approaches, which are simpler to implement [66, 67]. The method also requires long‐term wind datasets, which may limit applicability in data‐scarce regions. Furthermore, wind speeds were bias‐corrected at the standard 10 m reference height and subsequently extrapolated to 100 m to represent turbine hub heights, introducing additional uncertainty related to boundary layer assumptions.

While commonly used indicators such as wind power density and turbine‐specific capacity factors exist, this study focuses on the Weibull scale parameter and exceedance probabilities as physically consistent and data‐efficient proxies for wind potential. These indicators capture both the magnitude and persistence of wind speeds without relying on assumptions about specific turbine models or hub heights, ensuring comparability across scenarios.

Traditional GIS‐MCDM methods are more suited for short‐term decision‐making, where wind resource stability is assumed. However, for long‐term wind energy planning under climate change, the proposed framework provides a more robust and adaptive approach, ensuring that selected locations remain viable under future atmospheric conditions. By integrating variability, probabilistic behavior, and future projections, the framework supports more reliable long‐term wind energy planning.

Given the increasing impact of climate change on wind dynamics, future planning should prioritize methods that integrate long‐term projections and variability. The proposed approach enhances investment reliability by identifying sites with sustained wind potential over time.

## 4 Conclusion

This study presented a comprehensive assessment of wind energy potential across CA based on historical,1992–2021, and future, 2022–2100, climate conditions under SSP2–4.5 and SSP5–8.5 scenarios. Using a multi‐model ensemble of bias‐corrected CMIP6 projections and ERA5 reanalysis, we evaluated seasonal wind speed trends, fitted Weibull‐based wind speed distributions, calculated exceedance probabilities, and introduced a tier‐based classification system to assess the resilience of potential wind energy sites.

Historical results showed strengthening of wind speeds during spring and summer in northern countries, with slopes reaching +0.126 in Kyrgyzstan and +0.094 in Kazakhstan, while fall exhibited significant weakening, particularly in Tajikistan (−0.127) and Turkmenistan (−0.095). Future projections revealed widespread declines in wind speeds, especially under SSP5–8.5, with fall slopes dropping to −0.292 in Kazakhstan and −0.247 in Kyrgyzstan. Seasonal shifting patterns were identified, including a transition toward winter‐dominant wind seasons in parts of Kazakhstan and Uzbekistan. Weibull‐based analysis indicated modest reductions in the scale parameter, typically ranging from 0.1 to 0.3 m/s, particularly in southern regions, suggesting small decreases in mean wind speeds and slight increases in wind variability under future scenarios.

The developed tier classification highlighted strong regional contrasts. Kazakhstan emerges as the most stable country, with approximately 23.4% of sites ranked as Tier 1, while Kyrgyzstan has less than 2% Tier 1 sites. Southern countries, including Turkmenistan (88.2% Tier 5) and Tajikistan (57.1% Tier 5), were dominated by unstable or inconsistent locations, requiring detailed case‐by‐case evaluation. Furthermore, over 74% of sites across the region were projected to experience tier downgrades by mid‐century, reflecting growing climate scenario sensitivity, particularly during spring and fall.

Overall, the combined evidence across seasonal trends, variability metrics, Weibull analysis, and ranking‐based assessments consistently indicates a shift toward increased spatial heterogeneity and growing sensitivity of wind energy potential to climate change across CA.

These findings emphasize that relying solely on historical wind averages is insufficient for future wind energy development. Future planning must integrate seasonal shifts, probabilistic exceedance metrics, and scenario sensitivity into investment strategies. Prioritizing resilient Tier 1 sites while adopting adaptive, flexible approaches for vulnerable regions will be essential to ensuring sustainable, reliable wind energy systems across CA under evolving climate conditions.

From a policy perspective, these results highlight the need for coordinated regional strategies to ensure energy security under changing wind regimes. Integrating wind projections into national renewable‐energy roadmaps can support diversification of power generation and reduce dependency on fossil fuels. Strengthening cross‐border energy cooperation, modernizing grid interconnections, and combining wind with solar and hydropower resources would enhance system flexibility and resilience. Policymakers should also promote investment incentives, technology transfer, and adaptive infrastructure planning to mitigate climate‐related energy risks and accelerate progress toward national and regional renewable‐energy targets.

## Author Contributions

**Parya Broomandi**: writing – original draft, writing – review and editing, conceptualization, methodology, validation, formal analysis, investigation, and data curation. **Mehdi Bagheri**: writing – review and editing, conceptualization, methodology, validation, resources, project administration, and funding acquisition. **Sonny Irawan**: data curation, validation, supervision, review and editing, resources, project administration, and funding acquisition. **Aram Fathian**: formal analysis, data curation, and visualization. **Ali Mozhdehi Fard**: methodology, formal analysis, and data curation. **Dorna Gholamzade Ledari**: writing – original draft and data curation. **Michael Leuchner**: investigation, validation, review, and editing. **Jong Ryeol Kim**: resources, data curation, project administration, funding acquisition, review, and editing.

## Ethical Approval

The authors have nothing to report.

## Consent

The authors have nothing to report.

## Conflicts of Interest

The authors declare no conflicts of interest.

## Supporting information




**Supporting File**: gch270109‐sup‐0001‐SuppMat.docx.

## Data Availability

The data that support the findings of this study are available from the corresponding author upon reasonable request.
