# Supplementary material for: Climate‐Driven Shifts in Wind Energy Potential Across Central Asia Based on a Multi‐Model Ensemble and Tier‐Based Assessment
Source: Glob Chall. 2026 Sep 12;10(9):e00503. doi: 10.1002/gch2.202500503 (PMC13570334; doi:10.1002/gch2.202500503)
Supplement: Supplementary file 1 [file GCH2-10-e00503-s001.docx]

**Climate-Driven Shifts in Wind Energy Potential across Central Asia Based on a Multi-Model Ensemble and Tier-Based Assessment**

Parya Broomandi^1,2^, Mehdi Bagheri^2*^, Sonny Irawan^3^, Aram Fathian^4^, Ali Mozhdehi Fard^1^, Dorna Gholamzade Ledari^2^, Michael Leuchner^5^, Jong Ryeol Kim^1*^

^1^Department of Civil and Environmental Engineering, School of Engineering and Digital Sciences, Nazarbayev University, Kabanbay Batyr Ave. 53, Astana 010000 Kazakhstan.

^2^Department of Electrical and Computer Engineering, School of Engineering and Digital Sciences, Nazarbayev University, Kabanbay Batyr Ave. 53, Astana 010000 Kazakhstan.

^3^Department of Petroleum Engineering Department, School of Mining and Geoscience, Nazarbayev University, Kabanbay Batyr Ave. 53, Astana 010000 Kazakhstan.

^4^Water, Sediment, Hazards, and Earth-surface Dynamics (waterSHED) Lab, Department of Geoscience, University of Calgary, Calgary Alberta T2N 1N4, Canada.

^5^Physical Geography and Climatology, Department of Geography, RWTH Aachen University, Wüllnerstr. 5b, 52056 Aachen, Germany.

* Corresponding authors. Email: mehdi.bagheri@nu.edu.kz, and Phone: +7 (7172) 70-92-51.
 Email: jong.kim@nu.edu.kz, and Phone: +7 (7172) 70-91-36.

| **Table S1.** The list of GCM models used in the study ([https://www.ipcc.ch/report/ar6](https://www.ipcc.ch/report/ar6/wg1/downloads/report/IPCC_AR6_WGI_AnnexII.pdf)). | | |
| --- | --- | --- |
| MODELS | Resolution(km) and Number of Levels (L) | Institution/Country |
| ACCESS-CM2 | 140 km, 85 L | csiro-access csiro and Australia. res. council center of excellence for climate system science, Australia |
| CMCC-ESM2 | ~100 km, 30 L | Centro Euro-Mediterraneo sui Cambiamenti Climatici (CMCC), Italy |
| CESM2 | ~100 km, 32 L | NCAR, National Center for Atmospheric Research, USA |
| INM-CM5-0 | ~200 km, 73L | Institute for Numerical Mathematics(INM) , Russian Academy of Sciences (Russia) |
| HadGEM3-GC31-LL | ~250 km, 85 L | Met Office Hadley Centre (MOHC), United Kingdom |
| NorESM2-MM | ~100 km, 32 L | NorESM Climate modeling Consortium (NCC), Norway |
| IITM-ESM | ~200 km, 64 L | Centre for Climate Change Research, Indian Institute of Tropical Meteorology (CCCR-IITM), India |
| IPSL-CM6A-LR | ~250 km, 79 L | Institut Pierre-Simon Laplace (IPSL), France |
| KIOST-ESM | ~250 km, 32 L | Korea Institute of Ocean Science and Technology (KIOST), South Korea |
| CMCC-CM2-SR5 | ~100 km, 30 L | Centro Euro-Mediterraneo sui Cambiamenti Climatici (CMCC), Italy |
| GFDL-ESM4 | ~100 km, 49 L | National Oceanic and Atmospheric Administration Geophysical Fluid Dynamics Laboratory (NOAA-GFDL), United States |
| INM-CM4-8 | ~200 km, 21 L | Institute for Numerical Mathematics (INM), Russia |
| MIROC6 | 250 km, 40 L | MIROC Consortium JAMSTEC, AORI, NIES, R-CCS, Japan |
| MIROC-ES2L | 250 km, 40 L | MIROC Consortium JAMSTEC, AORI, NIES, R-CCS, Japan |
| MPI-ESM1-2-LR | 170 km, 47 L | MPI-M, Max Planck Institute for Meteorology, Germany |
| MRI-ESM2-0 | 100 km, 80 L | MRI, Meteorological Research Institute, Japan |
| BCC-CSM2-MR | ~100 km, 46 L | Beijing Climate Center (BCC), China |
| KACE-1-0-G | ~250 km, 85 L | National Institute of Meteorological Sciences/Korea Meteorological Administration (NIMS-KMA), South Korea |
| NorESM2-LM | ~250 km, 32 L | NorESM Climate modeling Consortium (NCC), Norway |
| UKESM1-0-LL | ~135 km, 85 L | Met Office Hadley Centre (MOHC) and Natural Environment Research Council (NERC), United Kingdom |

| **Table S2.** Selected highest- and lowest-ranked locations for wind energy suitability across CA under historical (1992–2021), near-future (2022–2051), and far-future (2071–2100) periods under SSP2–4.5 and SSP5–8.5 scenarios. | | | | | | |
| --- | --- | --- | --- | --- | --- | --- |
| **Historical (1992-2021)** | | | | | | |
| **Ranking (Top/Low)** | | **TM** | **KZ** | **TJ** | **KG** | **UZ** |
| **Top** | **1** | Turkmenba | Fort-Shevchenko | Khujand | Komsomol | Urganch |
|  | **2** | Atamyrat | Akshukyr | Isfisor | Aral | Nukus |
|  | **3** | Balkanabat | Yerkinkala | Ghafurov | Blagoveshchenskoye | Bukhara |
|  | **4** | Yoloten | Karaton | Lohut | Karakol | Kogon Shahri |
|  | **5** | Dasoguz | Atyrau | Ghulakandoz | Alga | Termiz |
| **Low** | **1** | Kaka | Kaskelen | Vanj | Sargata | Iskandar |
|  | **2** | Abadan | Maleyevsk | Rushon | Uch-Terek | Shahrixon |
|  | **3** | Ashgabat | Altay | Almasi | Kyzyl-Bulak | Buloqboshi |
|  | **4** | Anew | Pervomayka | Chorbog | Orozbekov | Marhamat |
|  | **5** | Magtymguly | Granitogorsk | Varzob | Kanysh-Kyya | Xojaobod |
| **Near-Future (2022-2051) SSP2–4.5** | | | | | | |
| **Ranking (Top/Low)** | | **TM** | **KZ** | **TJ** | **KG** | **UZ** |
| **Top** | **1** | Turkmenba | Fort-Shevchenko | Khujand | Komsomol | Kogon Shahri |
|  | **2** | Atamyrat | Akshukyr | Isfisor | Aral | Bukhara |
|  | **3** | Yoloten | Yerkinkala | Ghafurov | Blagoveshchenskoye | Urganch |
|  | **4** | Balkanabat | Balykshi | Lohut | Karakol | Nukus |
|  | **5** | Turkmenabat | Atyrau | Ghulakandoz | Prigorodnoye | Termiz |
| **Low** | **1** | Kaka | Altay | Rushon | Cholpon-Ata | Buloqboshi |
|  | **2** | Abadan | Maleyevsk | Jirghatol | Kanysh-Kyya | Marhamat |
|  | **3** | Ashgabat | Kaskelen | Vanj | Bosteri | Xojaobod |
|  | **4** | Anew | Burunday | Khorugh | Chaek | Poytug |
|  | **5** | Magtymguly | Pervorossiyskoye | Roshtqala | Kazarman | Paxtaobod |
| **Far-Future (2071-2100) SSP2–4.5** | | | | | | |
| **Ranking (Top/Low)** | | **TM** | **KZ** | **TJ** | **KG** | **UZ** |
| **Top** | **1** | Turkmenba | Fort-Shevchenko | Khujand | Komsomol | Urganch |
|  | **2** | Atamyrat | Akshukyr | Isfisor | Aral | Kogon Shahri |
|  | **3** | Yoloten | Karaton | Ghafurov | Blagoveshchenskoye | Bukhara |
|  | **4** | Balkanabat | Yerkinkala | Lohut | Karakol | Nukus |
|  | **5** | Turkmenabat | Beyneu | Proletar | Alga | Termiz |
| **Low** | **1** | Kaka | Maleyevsk | Rushon | Cholpon-Ata | Buloqboshi |
|  | **2** | Abadan | Altay | Jirghatol | Kanysh-Kyya | Marhamat |
|  | **3** | Ashgabat | Kaskelen | Vanj | Bosteri | Xojaobod |
|  | **4** | Anew | Burunday | Khorugh | Chaek | Poytug |
|  | **5** | Magtymguly | Pervorossiyskoye | Almasi | Kazarman | Paxtaobod |
| **Near-Future (2022-2051) SSP5–8.5** | | | | | | |
| **Ranking (Top/Low)** | | **TM** | **KZ** | **TJ** | **KG** | **UZ** |
| **Top** | **1** | Turkmenba | Fort-Shevchenko | Khujand | Komsomol | Urganch |
|  | **2** | Atamyrat | Akshukyr | Isfisor | Aral | Kogon Shahri |
|  | **3** | Yoloten | Karaton | Ghafurov | Blagoveshchenskoye | Bukhara |
|  | **4** | Balkanabat | Yerkinkala | Lohut | Karakol | Nukus |
|  | **5** | Turkmenabat | Beyneu | Ghulakandoz | Prigorodnoye | Termiz |
| **Low** | **1** | Kaka | Altay | Jirghatol | Cholpon-Ata | Buloqboshi |
|  | **2** | Abadan | Maleyevsk | Rushon | Kanysh-Kyya | Marhamat |
|  | **3** | Ashgabat | Kaskelen | Vanj | Chaek | Xojaobod |
|  | **4** | Anew | Burunday | Khorugh | Bosteri | Poytug |
|  | **5** | Magtymguly | Pervorossiyskoye | Roshtqala | Kazarman | Paxtaobod |
| Far-Future (2071-2100) SSP5–8.5 | | | | | | |
| **Ranking (Top/Low)** | | **TM** | **KZ** | **TJ** | **KG** | **UZ** |
| **Top** | **1** | Turkmenba | Fort-Shevchenko | Khujand | Komsomol | Urganch |
|  | **2** | Atamyrat | Akshukyr | Isfisor | Aral | Nukus |
|  | **3** | Yoloten | Karaton | Ghafurov | Blagoveshchenskoye | Kogon Shahri |
|  | **4** | Balkanabat | Beyneu | Lohut | Karakol | Bukhara |
|  | **5** | Turkmenabat | Koschagyl | Proletar | Alga | Termiz |
| **Low** | **1** | Kaka | Altay | Rushon | Cholpon-Ata | Buloqboshi |
|  | **2** | Abadan | Maleyevsk | Vanj | Kanysh-Kyya | Marhamat |
|  | **3** | Ashgabat | Kaskelen | Jirghatol | Bosteri | Xojaobod |
|  | **4** | Anew | Burunday | Khorugh | Chaek | Poytug |
|  | **5** | Magtymguly | Kegen | Almasi | Kazarman | Paxtaobod |

**Fig. S1.** Projected changes in the frequency of marginal wind conditions (near turbine cut-in thresholds) across Central Asian countries under SSP2–4.5 and SSP5–8.5 scenarios for near- and far-future periods.

**Fig. S2.** Projected changes in the frequency of moderate wind conditions (low-efficiency operational range) across Central Asian countries under SSP2–4.5 and SSP5–8.5 scenarios for near- and far-future periods.

**Fig. S3.** Projected changes in the frequency of optimal wind conditions (high-efficiency operational range) across Central Asian countries under SSP2–4.5 and SSP5–8.5 scenarios for near- and far-future periods.

**Fig. S4.** Seasonal wind dominance in Tajikistan across historical, near-future (2022–2051), and far-future (2071–2100) periods under SSP2–4.5 and SSP5–8.5 scenarios.

**Fig. S5.** Seasonal wind dominance in Turkmenistan across historical, near-future (2022–2051), and far-future (2071–2100) periods under SSP2–4.5 and SSP5–8.5 scenarios.

**Fig. S6.** Seasonal wind dominance in Uzbekistan across historical, near-future (2022–2051), and far-future (2071–2100) periods under SSP2–4.5 and SSP5–8.5 scenarios.

**Fig. S7.** Seasonal wind dominance in Kyrgyzstan across historical, near-future (2022–2051), and far-future (2071–2100) periods under SSP2–4.5 and SSP5–8.5 scenarios.

**Fig. S8.** Dominant season transition matrices for Tajikistan under SSP2–4.5 and SSP5–8.5 scenarios for near-future (2022–2051) and far-future (2071–2100) periods.

**Fig. S9.** Dominant season transition matrices for Turkmenistan under SSP2–4.5 and SSP5–8.5 scenarios for near-future (2022–2051) and far-future (2071–2100) periods.

**Fig. S10.** Dominant season transition matrices for Uzbekistan under SSP2–4.5 and SSP5–8.5 scenarios for near-future (2022–2051) and far-future (2071–2100) periods.

**Fig. S11.** Dominant season transition matrices for Kyrgyzstan under SSP2–4.5 and SSP5–8.5 scenarios for near-future (2022–2051) and far-future (2071–2100) periods.

**Fig. S12.** Changes (%) in location-based wind energy ranking scores in Uzbekistan relative to the historical baseline, under SSP2–4.5 and SSP5–8.5 scenarios.

**Fig. S13.** Changes (%) in location-based wind energy ranking scores in Tajikistan relative to the historical baseline, under SSP2–4.5 and SSP5–8.5 scenarios.

**Fig. S14.** Changes (%) in location-based wind energy ranking scores in Turkmenistan relative to the historical baseline, under SSP2–4.5 and SSP5–8.5 scenarios.

**Fig. S15.** Changes (%) in location-based wind energy ranking scores in Kyrgyzstan relative to the historical baseline, under SSP2–4.5 and SSP5–8.5 scenarios.
